# IκB-ζ signaling promotes chondrocyte inflammatory phenotype, senescence, and erosive joint pathology

**DOI:** 10.1038/s41413-021-00183-9

**Published:** 2022-02-11

**Authors:** Manoj Arra, Gaurav Swarnkar, Yael Alippe, Gabriel Mbalaviele, Yousef Abu-Amer

**Affiliations:** 1 Department of Orthopaedic Surgery and Cell Biology & Physiology, Washington University School of Medicine, St. Louis, MO 63110 US; 2 Bone and Mineral Division, Department of Medicine, Washington University School of Medicine, St. Louis, MO 63110 US; 3 Shriners Hospital for Children, St. Louis, MO 63110 US

**Keywords:** Metabolism, Pathogenesis

## Abstract

Osteoarthritis is a joint disease characterized by a poorly-defined inflammatory response that does not encompass a massive immune cell infiltration yet contributes to cartilage degradation and loss of joint mobility, suggesting a chondrocyte intrinsic inflammatory response. Using primary chondrocytes from joints of osteoarthritic mice and patients, we first show that these cells express ample pro-inflammatory markers and RANKL in an NF-κB dependent manner. The inflammatory phenotype of chondrocytes was recapitulated by exposure of chondrocytes to IL-1β and bone particles, which were used to model bone matrix breakdown products revealed to be present in synovial fluid of OA patients, albeit their role was not defined. We further show that bone particles and IL-1β can promote senescent and apoptotic changes in primary chondrocytes due to oxidative stress from various cellular sources such as the mitochondria. Finally, we provide evidence that inflammation, oxidative stress and senescence converge upon IκB-ζ, the principal mediator downstream of NF-κB, which regulates expression of RANKL, inflammatory, catabolic, and SASP genes. Overall, this work highlights the capacity and mechanisms by which inflammatory cues, primarily joint degradation products, i.e., bone matrix particles in concert with IL-1β in the joint microenvironment, program chondrocytes into an “inflammatory phenotype” which inflects local tissue damage.

## Introduction

Osteoarthritis (OA) is the most prevalent joint disease worldwide, causing pain, decreased quality of life, and reduced productivity in those affected by this condition.^1–3^ However, current OA treatment regimens do not contain any disease-modifying therapeutics but instead rely upon symptom-reducing compounds, though promising advancements have been made in the field. Given the prevalence of this disease, novel targets and therapeutics are greatly needed to combat the impact of OA on patients and society.

While OA was traditionally considered as non-inflammatory arthritis, extensive work has displayed the role of inflammation in OA.^4–7^ Inflammation is induced by many processes, ranging from mechanical stress to stimulation by inflammatory cytokines, all of which play a role in the initiation and propagation of this disease.^8,9^ It is likely that OA is induced by various acute inflammatory insults throughout life, such as mechanical injury, which then contribute to a sustained chronic, inflammatory state as patients grow older. The response of articular chondrocytes to these inflammatory stimuli contributes to the development and progression of OA by promoting the expression of catabolic factors that break down ECM that normally gives cartilage its joint protective properties. Furthermore, the role of senescent chondrocytes in articular cartilage as a source of pro-inflammatory factors has been highlighted in recent years,^10–13^ primarily due to the production of senescence-associated secretory phenotype (SASP) factors that are pathogenic. This can lead to a positive feedback cycle of chronic inflammation through autocrine and paracrine effects. Although it is still unclear what drives chondrocyte senescence, some possibilities include factors such as oxidative stress, microRNAs, and inflammatory pathways.^14–16^ Oxidative and nitrosative stresses have especially gained interest because they can arise from multiple components within the cell and are intricately related to inflammation and cellular metabolism.^17^ Reactive oxygen species such as superoxide can damage DNA, proteins, and lipids to alter cellular physiology and promote pathology. It is predicted that prevention of senescence or reduction of SASP expression from senescent chondrocytes, which overlaps with the inflammatory response, may be important for reducing the chronic inflammatory state of OA joints and prevent OA progression.^18^

The role of cartilage and bone degradative by-products in OA disease pathology is also an area that requires further research. The presence of calcium crystals and other by-products of bone and ECM degradation has been noted in the synovial fluid of OA joints, with some groups suggesting that these particles are pro-pathogenic while others suggest that they are merely by-products of disease.^19–21^ The source of these particles is still unknown, though it is predicted to be from a combination of cartilage calcification by hypertrophic chondrocytes, bone erosion of juxta-articular regions, or bone-on-bone erosions.^22^ Even in the setting of bone-on-bone erosion or internal knee injuries that release bone fragments, cartilage remains present in the joint, and understanding the function of these complex bone particles (BP) on the remaining joint health needs to be elucidated, especially if future therapies aim to focus on cartilage regeneration. One of the major findings in OA patients is the alteration of subchondral bone physiology and structure, leading to the prediction that interaction between articular cartilage and subchondral bone cells is important for OA pathogenesis.^23–25^ Subchondral bone sclerosis and osteophyte formation late in OA is characteristic of the disease, though it is unclear if early OA has similar findings and should be further explored. It is possible that altered catabolism of subchondral bone may be a contributor to the presence of crystals in the synovial space, especially by osteoclast activity promoted by chondrocytes.^26^ Previous reports indicate that matrix fragments and crystals can be pathological, with some animal studies suggesting that these particles contribute to the chronic inflammatory state of OA synovial joints.^27–31^

The goal of this work is to explore the effects of pathogenic molecular entities and associated inflammatory signals, such as BP and IL-1β, on chondrocytes and the downstream mediators involved. In this work, we display that chondrocytes can support osteoclast formation in response to inflammatory stimuli through increased Rankl production. Furthermore, we show that bone matrix particles are highly pro-inflammatory and can synergize with inflammatory cytokines to drive catabolic enzyme expression in an NF-κB dependent manner. We also display that inflammatory stimuli can promote senescent and apoptotic changes. Finally, we show that BP and IL-1β act via IκB-ζ to promote SASP expression in a manner dependent upon oxidative and nitrosative stresses.

## Results

### OA joints have increased osteoclast activity and Rankl production by chondrocytes

**Fig. 1:**
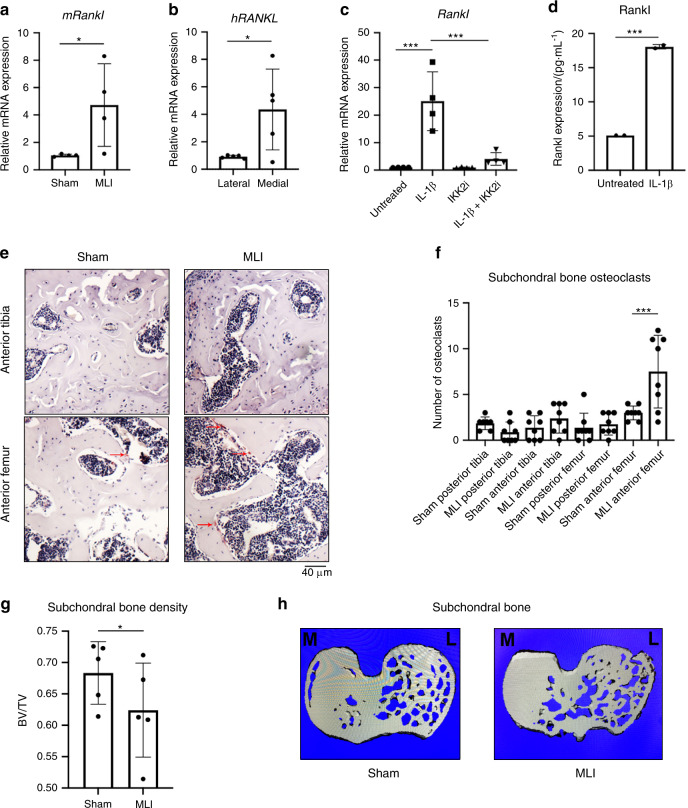
Chondrocytes produce Rankl under inflammation. **a** MLI surgery was performed on 12-week-old mice. After 4 weeks, articular cartilage was isolated from sham and MLI knee joints and gene expression of *Rankl* was measured (*n* = 4, *P = 0.050). Data are mean ± SEM. **b** Medial and lateral knee articular cartilage was isolated from patients undergoing TKA with medial compartment OA. Gene expression of *human RANKL* comparing more damaged medial cartilage to lateral cartilage (*n* = 5, *P = 0.031). Data are mean ± SEM. **c** Primary chondrocytes were cultured with IL-1β (10 ng·mL^−^^1^) ± SC-514 (10 μmol·L^−1^) for 24 h. *Rankl* gene expression was measured (Untreated vs IL-1β ***P = 0.000 2, IL-1β vs IL-1β + IKK2i ***P = 0.000 7), with data representing mean ± SEM of *n* = 4 independent experiments. **d** Rankl protein expression in IL-1β treated (24 h) chondrocytes. Data are mean ± SD for *n* = 2 representative replicates (***P = 0.003 3). **e** Two weeks post MLI, knee joints were fixed and sectioned for TRAP staining to identify osteoclasts in the subchondral region (arrows). **f** TRAP positive cells were counted in anterior and posterior compartments of femur and tibia in MLI and Sham joints (*n* = 8). (Sham Anterior Femur vs MLI Anterior Femur ***P = 0.002). Bars represent mean ± S.D. **g** MLI and sham surgeries were performed on right and left mouse knees, respectively. After 2 weeks, mice were sacrificed and joints were harvested and fixed. Bone volume/total volume of subchondral region was measured by uCT in same region of each mouse tibia (*n* = 5 mice) (*P = 0.036 5). **h** Representative image of subchondral slice from sham and MLI mouse joints displaying decreased bone volume (M and L indicate medial and lateral condyle, respectively)

It has been noted that joints likely undergo erosive changes during the early stages of OA, followed by subchondral sclerosis in advanced stages.^32^ We surmised that the local joint microenvironment supports bone and cartilage erosion through elevated osteoclast activity, contributing to subchondral changes. Hence, we set out to determine if OA chondrocytes in humans and murine models display increased receptor activator of NF-κB ligand (Rankl) expression, the critical factor required for osteoclastogenesis. We utilized the well-established meniscal ligamentous injury (MLI) model to provoke experimental OA in mice^33^ and observed that cartilage from MLI joints after surgery had elevated levels of *Rankl (Tnfsf11)* mRNA expression compared to sham surgery joints (Fig. 1a). We also observed similar changes in human OA cartilage when comparing more damaged medial cartilage to relatively healthy lateral cartilage in patients with medial compartment OA (Fig. 1b). Since we have recently shown that inflammation is a significant driver of OA joint degradation,^34^ we treated mouse-derived chondrocytes with IL-1β, a major inflammatory mediator and marker of OA. We observed that exposure of chondrocytes to IL-1β significantly increased the expression of *Rankl* mRNA and Rankl protein secretion (Fig. 1c, d). Furthermore, we have previously demonstrated in articular chondrocytes that NF-κB activation, the principle inflammatory response pathway, is a critical component of OA pathogenesis^34^ and predicted that the increase in Rankl expression is mediated via NF-κB activation. Confirming this proposition, we show that NF-κB activation using the constitutively active form of IKK2 (IKK2ca) also drives *Rankl* expression mimicking inflammatory stimuli (Supplementary Fig. S1a). This was further validated using the IKK2 inhibitor, SC-514, which reduced *Rankl* expression in IL-1β-treated chondrocytes (Fig. 1c). Since osteoprotegerin (Opg) is also an important counterpart to Rankl in modulating osteoclastogenesis by negatively regulating osteoclast formation, we sought to determine the *Rankl:Opg* ratio.^35^ We observed that IL-1β treatment decreased *Opg* mRNA expression (Supplementary Fig. S1b) and greatly increased the *Rankl:Opg* ratio but the application of IKK2 inhibitor reduced the *Rankl:Opg* ratio through both reduction in *Rankl* and increase in *Opg* mRNA expression (Supplementary Fig. S1c).

Consistent with these observations, we predicted that chondrocytes may be an important source of Rankl near damaged cartilage. We first tested whether the presence of Rankl in the synovial fluid can promote subchondral osteoclast formation. Recombinant Rankl was injected into the synovial space by intra-articular injection, with PBS injection used as control. Rankl injection into the synovial space led to a dramatic increase in osteoclasts in the subchondral region (Supplementary Fig. S1d, e). We then show that post-surgery MLI joints displayed increased tartrate-resistant acid phosphatase (TRAP)-positive staining in the anterior femoral subchondral bone region compared to contralateral sham joints, indicating increased osteoclast activity and altered bone resorption (Fig. 1e, f). However, we did not observe statistically significant differences in osteoclast number in the tibial regions or posterior femoral regions. When analyzed by μCT, MLI joints in mice (Supplementary Fig. S1g) at 2 weeks post-surgery have increased bone erosion and subchondral bone loss compared to sham joints as measured by subchondral bone volume (Fig. 1g, h, Supplementary Fig. S1f). We also utilized the Aggrecan-ERT2-cre IKK2ca (Acan^IKK2ca^) mouse model, which displays IKK2 activation in articular chondrocytes, that we have previously shown can drive cartilage degradation and mimic an OA-like phenotype.^34^ These joints also have decreased joint spacing and blunting of the bony surfaces in the subchondral region similar to post-traumatic OA even in the absence of mechanical injury (Supplementary Fig. S1i). This suggests that chondrocyte-specific NF-κB activity can cause bone degradation and modify the subchondral region, potentially via Rankl expression and altered mechanical properties of the joint due to cartilage degradation.

**Fig. 2:**
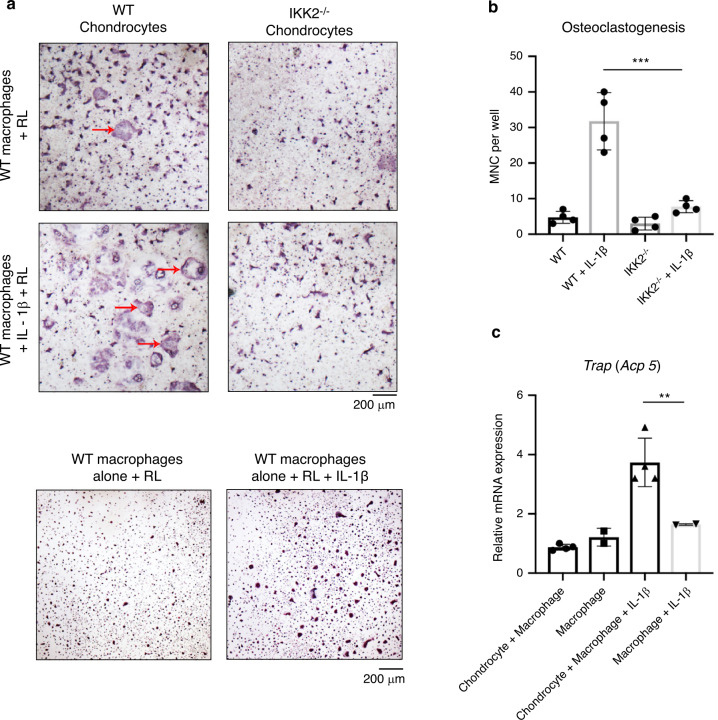
Chondrocytes support osteoclastogenesis under inflammatory conditions. **a** Primary IKK2^f/f^ chondrocytes were isolated and infected with adenoviral-GFP or adenoviral-cre to delete IKK2. Bone marrow macrophages were then added to the culture. Permissive levels of Rankl (5 ng·mL^−1^) was added ± IL-1β (10 ng·mL^−1^) for 3 days. Cultures were fixed and stained for TRAP positive osteoclasts (red arrows). Macrophages alone cultures are displayed as control. **b** Number of large, multi nucleated cells (MNC) in macrophage-chondrocyte co-culture were quantified by counting. (WT + IL-1β vs IKK2^−/−^ + IL-1β ***P = 0.000 2, *n* = 4 wells from representative experiment). Bars represent mean ± S.D. **c** mRNA was isolated from co-cultures of chondrocytes and macrophages ± IL-1β. Gene expression analysis was performed for TRAP (**P = 0.001 7, *n* = 4). Bars represent mean ± S.D.

To substantiate our observations, we sought to determine if chondrocytes can directly promote osteoclast formation and regulate bone degradation. We utilized a chondrocyte-macrophage co-culture system to allow for the interaction of chondrocyte-secreted factors with bone marrow macrophages to determine the effect on osteoclast differentiation. At basal conditions, chondrocytes co-cultured with macrophages did not increase the presence of TRAP positive cells compared to macrophages in culture alone since chondrocytes produce significant amounts of Opg under physiologic conditions.^26^ Given that chondrocytes treated with IL-1β had increased expression of Rankl, we then asked if under inflammatory conditions, chondrocytes could support and promote osteoclastogenesis. We show that macrophage-chondrocyte co-cultures treated with IL-1β had increased TRAP positive cells compared to those without IL-1β in the presence of permissive levels of exogenous Rankl, which was added to prime commitment of monocyte/macrophage into OC precursors (Fig. 2a, b). We confirmed this to be a chondrocyte-mediated effect by comparing co-cultures using IKK2 deficient chondrocytes treated with IL-1β and Rankl, which displayed significantly less TRAP positive multi nucleated cells (Fig. 2a, b; right panels). This finding suggests that IKK2 mediates the IL-1β inflammatory response of chondrocytes to produce Rankl and enhance osteoclastogenesis (Fig. 2a, b). Mirroring these findings, we observed that chondrocyte-macrophage co-cultures had significantly higher TRAP expression when treated with IL-1β compared to macrophages treated with IL-1β alone, suggesting that chondrocytes can support macrophage differentiation into osteoclasts under inflammatory conditions (Fig. 2c).

### Bone tissue particles are potent pro-inflammatory molecules that act in an NF-κB dependent manner

**Fig. 3:**
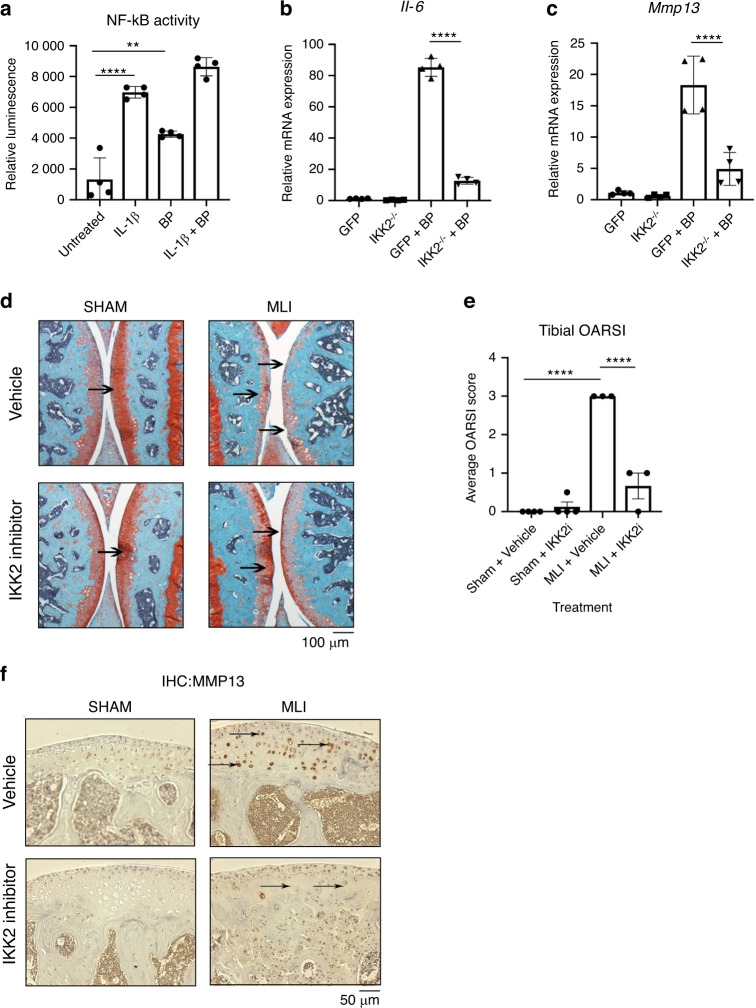
Bone particles drive the production of catabolic genes via NF-κB. **a** Chondrocytes from NF-κB luciferase reporter mice were cultured with BP or IL-1β for 24 h. Luciferase assay was performed to measure NF-κB activation (Untreated vs IL-1β ****P < 0.000 1, Untreated vs BP **P = 0.001, *n* = 4 independent replicates). Results are mean ± SD from one of three representative experiments. **b**, **c** IKK2^−/−^ chondrocytes were transduced with adeno-GFP or adeno-cre. Cells were then cultured with BP for 24 h. *Il-6* and *Mmp13* gene expression was measured (*IL-6*: GFP + BP vs IKK2^−/−^ + BP ^****^P < 0.000 1. *MMP13*: GFP + BP vs IKK2^−/−^ + BP ****P < 0.000 1). Results are mean ± SD for *n* = 4 replicates. **d** MLI surgery was performed on 12-week-old mice. IKK2 inhibitor was injected I.P. for 6 weeks and joints were harvested for histology to perform safranin-O staining, with most damage to tibial surface (arrows). Representative images are displayed. **e** Blinded OARSI scoring was performed to grade OA severity on the tibial surface (Sham + Vehicle vs MLI + Vehicle ****P < 0.000 1, MLI + Vehicle vs MLI + IKK2i ****P < 0.000 1). Bars represent mean ± SEM. from *n* = 4 vehicle treated and *n* = 3 inhibitor treated mice. **f** IHC was performed for Mmp13 in articular cartilage of joint sections under the same conditions. Representative images are displayed

We then proposed that joint pathology is exacerbated by cartilage and bone mineral particles, released into the synovial space from various factors such as cartilage calcification, osteoclast resorption, and mechanical cartilage-bone erosion, which could act as pro-inflammatory mediators. These inert particles likely act in a pro-inflammatory manner to drive cartilage degradation based on findings from prior work studying inert crystals.^27,36^ Given the principal role of NF-κB as the major inflammatory response pathway, we predicted that bone matrix particles would activate this pathway. Since mineralized bone degradation products cannot be isolated from the joint fluid of mouse joints due to the small volume, we used BP as surrogates for proof-of-concept studies. BP were generated by grinding the cortical surface of porcine long bones, rinsed repeatedly in sterile PBS, and stored in 70% ethanol. These BP were then washed again with PBS before addition to cultures. We established that, similar to IL-1β, treatment of chondrocytes with BP potently activates NF-κB (Fig. 3a). BP were also able to activate expression of pathological inflammatory response genes including IL-6 and MMP13 (Fig. 3b, c). Furthermore, we show that BP are able to synergize with constitutively active IKK2 (IKK2ca) to increase the expression of *Il-6*, *Mmp13* and *Adamts4* (Supplementary Fig. S2a–c) akin to exacerbated disease conditions, relative to IKK2ca alone. To substantiate our claim that BP act via NF-κB, we tested if inhibition of NF-κB would be sufficient to prevent the inflammatory response induced by BP. We showed that genetic ablation of IKK2, the major NF-κB activating kinase, in chondrocytes led to significant reduction in inflammatory gene expression with BP treatment (Fig. 3b, c). We also utilized a kinase-dead form of IKK2 (IKK2-KD) that has a dominant-negative effect to show that it was able to prevent the inflammatory response induced by BP by preventing signal transduction through IKK2 (Supplementary Fig. S2d–f). Overall, these results suggest that BP can promote a strong inflammatory response causing catabolic changes in an NF-κB dependent manner.

Given the potent role of NF-κB in the IL-1β and BP inflammatory response, we sought to define if the anti-inflammatory effect of IKK2 inhibition observed in vitro could be translated in vivo to protect against OA. We injected mice with IKK2 inhibitor (IKK2i) systemically for 6 weeks post MLI surgery and observed significant increase in cartilage integrity and improved OARSI scoring in MLI joints of IKK2i-treated animals (Fig. 3d, e). In addition, there was significantly decreased staining for MMP13 in the articular cartilage of IKK2i-treated MLI joints, indicating decreased catabolic activity (Fig. 3f; arrows).

### Bone particles activate NLRP3 inflammasome and IκB-ζ in chondrocytes

**Fig. 4:**
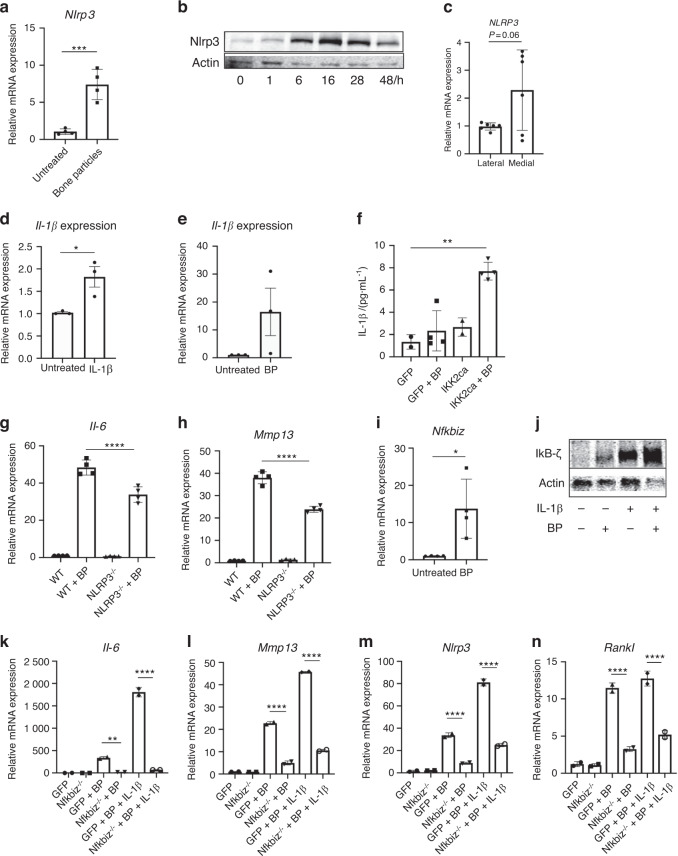
Bone particles promote inflammation via NLRP3 and IκB-ζ activity. **a** Primary chondrocytes were cultured with BP for 24 h and *Nlrp3* gene expression was measured (***P = 0.000 9). Bars represent mean ± SEM from *n* = 4 independent experiments. **b** Immunoblotting was performed for Nlrp3 protein using chondrocytes lysates under BP treatment time course, with actin loading used as control. The representative image is shown. **c** Medial and lateral knee articular cartilage was isolated from patients undergoing TKA with medial compartment OA. Gene expression analysis was performed for *NLRP3*. Bars represent mean ± S.D. for *n* = 6 samples. **d**, **e** Cells were treated with IL-1β or BP for 24 h and IL-1β gene expression was measured. Bars are mean ± SD from three separate experiments. (*P = 0.024 8). **f** Cells were retrovirally transduced with GFP or IKK2ca, then treated with bone particles for 24 h. Supernatant was collected and ELISA was performed for IL-1β levels (GFP vs IKK2ca + BP **P = 0.001 8, *n* = 4 replicates). **g**, **h** Chondrocytes isolated from WT or Nlrp3-deficient mice and treated with BP for 24 h. *Il-6* and *Mmp13* gene expression measured by qPCR (*IL-6*: WT + BP vs *Nlrp3*^−/−^ + BP ****P < 0.000 1, *Mmp*13: WT + BP vs *Nlrp3*^−/−^ + BP ^****^P < 0.000 1). Bars are mean ± SD for *n* = 4 replicates. **i** Primary chondrocytes were treated with BP for 24 h. *Nfkbiz* gene expression was measured by qPCR (*P = 0.018 7). Bars are mean ± SEM for *n* = 4 independent experiments. **j** Chondrocytes were exposed to IL-1β and/or bone particles. Lysates were collected and Immunoblotting was performed for IκB-ζ. Representative immunoblot is shown. **k**–**n** *Nfkbiz* flox chondrocytes were transduced with adeno-GFP (WT) or adeno-cre (*Nfkbiz*^−/−^). Cells were then treated with BP ± IL-1β. Gene expression of *Il-6*, *Mmp13*, *Nlrp3* and *Rankl* was measured by qPCR (*IL-6*: GFP + BP vs *Nfkbiz*^−/−^ + BP **P = 0.002 9, GFP^+^ BP + IL-1β vs *Nfkbiz*^−/−^ + BP + IL-1β ****P < 0.000 1. *Mmp13*: GFP + BP vs *Nfkbiz*^−/−^ + BP ****P < 0.000 1, GFP + BP + IL-1β vs *Nfkbiz*^−/−^ + BP + IL-1β ****P < 0.000 1. *Nlrp3*: GFP + BP vs *Nfkbiz*^−/−^ + BP ****P < 0.000 1, GFP + BP + IL-1β vs *Nfkbiz*^−/−^ + BP^+^ IL-1β ****P < 0.000 1. *Rankl*: GFP + BP vs *Nfkbiz*^−/−^ + BP ^****^P < 0.000 1, GFP + BP + IL-1β vs *Nfkbiz*^−/−^ + BP + IL-1β ****P < 0.000 1). Bars represent mean ± S.D. from one representative experiment out of three

We sought to further characterize the mechanism(s) underlying the response to BP exposure to identify potential therapeutic targets. Given that BP are not a signaling protein per se, we suspected that they may act as damage-associated molecular pattern (DAMP) molecules to stimulate the expression of inflammatory genes including Nlrp3, which assembles an inflammasome involved in the maturation of IL-1β, as well as the regulation of various intracellular processes such as apoptosis.^37^ We observed that BP treatment increases expression of Nlrp3 at both the gene and protein level (Fig. 4a, b). Furthermore, damaged human cartilage has elevated levels of *NLRP3* gene expression compared to healthier cartilage (Fig. 4c), suggesting that the NLRP3 inflammasome may be involved in the human disease.

We then displayed that IL-1β and BP treatment can both drive gene expression of IL-1β itself (Fig. 4d, e). The ability of IL-1β to induce its own expression suggests a feedforward pro-inflammatory mechanism by which chondrocytes can propagate inflammation in a paracrine or even autocrine manner. In agreement with our hypothesis that BP can act as DAMP signals that promote Nlrp3 activity, we observed that BP treatment of chondrocytes expressing IKK2ca significantly increased chondrocyte secretion of IL-1β, whereas individual treatment of chondrocytes with BP or IKK2ca expression alone is not a potent activator of IL-1β secretion (Fig. 4f). This suggests that NF-κB activation likely acts as a primary signal to promote Nlrp3 expression while BP act as the secondary signal for Nlrp3 assembly and activation. We also observe that *Nlrp3*-deficient chondrocytes have reduced *Il-6* and *Mmp13* expression upon bone particle treatment (Fig. 4g, h).

However, given that the inhibition of inflammatory gene expression resulting from Nlrp3 deletion was only partial, we sought to identify a factor further upstream that can be a more potent activator of this inflammatory response. Our previous work has displayed that incubation of chondrocytes with IL-1β increased gene expression of *Nfkbiz*,^34^, which encodes for IκB-ζ, a pro-inflammatory mediator.^38^ Furthermore, we have recently shown that MLI joints in mice and damaged cartilage from humans have elevated expression of *Nfkbiz* in the articular cartilage.^29^ Our work, and that of others, has displayed that IκB-ζ is a prominent pro-inflammatory secretome in OA chondrocytes and is necessary for the expression of various inflammatory and catabolic genes.^39^ To this end, treatment of chondrocytes with BP increased the expression of *Nfkbiz* at the gene level and IκB-ζ at the protein level (Fig. 4i, j). Combined BP and IL-1β treatment further increased protein levels of IκB-ζ (Fig. 4j). Supporting its direct role in this inflammatory response, treatment of *Nfkbiz*^*−/−*^ chondrocytes with BP failed to elicit a meaningful inflammatory response compared to wild type chondrocytes, even in the presence of an intact NF-κB signaling pathway (Fig. 4k, l). In addition, we observed that deletion of IκB-ζ decreased expression of *Nlrp3* (Fig. 4m), indicating that IκB-ζ is upstream of inflammasome expression and activation. We also observed that deletion of IκB-ζ decreased the expression of *Rankl* by BP or IL-1β-stimulated chondrocytes (Fig. 4n). Collectively, these findings suggest that under pathologic conditions, IκB-ζ appears to centrally control the expression of inflammatory, catabolic and osteoclastogenic factors.

### Inflammatory stimuli promote chondrocyte senescence

Recent reports have proposed that chondrocyte senescence plays a crucial part in OA pathogenesis^11,40,41^ and we suspected that inflammatory stimuli may contribute to chondrocyte senescent transformation. Thus, we screened RNA sequencing dataset from IL-1β-treated chondrocytes to determine how inflammatory stimuli affect gene expression, wherein we observed that apoptotic and senescence pathways are significantly altered (Table 1).

**Table 1:** Chondrocytes were treated with IL-1β for 24 h. RNA sequencing was performed and significantly altered pathways are displayed

Pathway	*P-*value
Regulation of cell proliferation	1.55E-13
Cellular response to DNA damage stimulus	1.23E-12
Positive regulation of apoptotic process	2.63E-10
Apoptotic process	8.49E-09
Intrinsic apoptotic signaling pathway in response to DNA damage	1.17E-06
Regulation of apoptotic process	3.66E-06
Intrinsic apoptotic signaling pathway in response to oxidative stress	8.66E-05
Extrinsic apoptotic signaling pathway	2.46E-04
Positive regulation of cell death	1.63E-03
Positive regulation of cell cycle arrest	3.54E-03
Apoptotic mitochondrial changes	8.60E-03
Cellular senescence	1.49E-02
Replicative senescence	3.90E-02

**Fig. 5:**
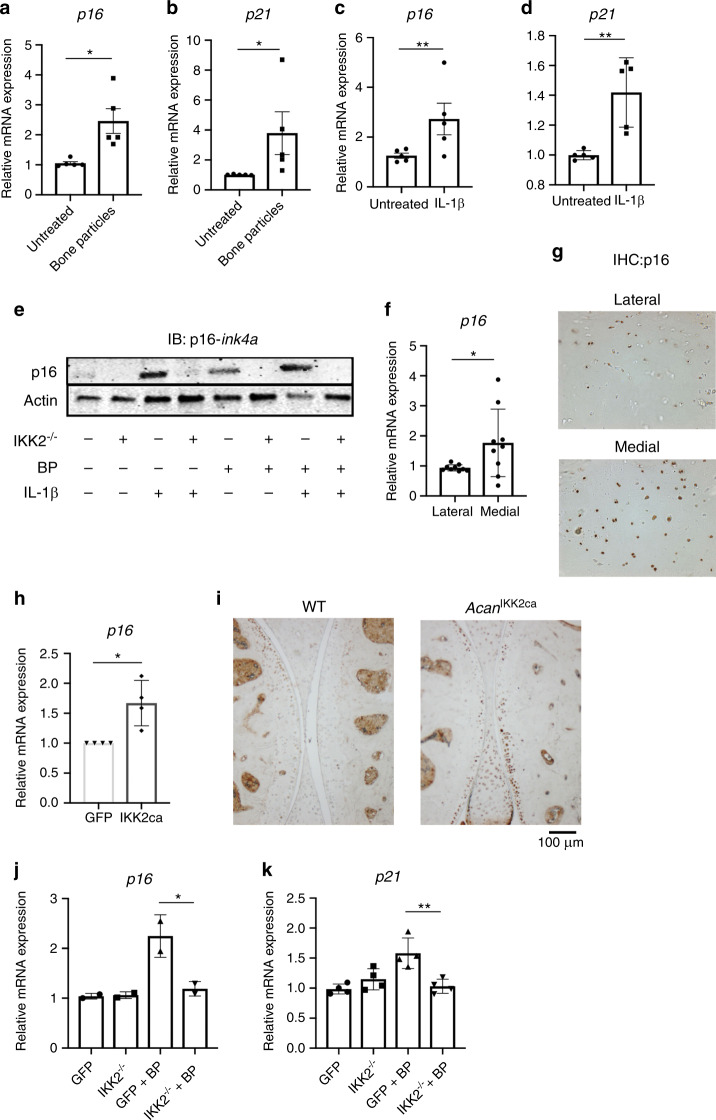
Inflammatory mediators promote senescent changes in chondrocytes. **a**–**d** Chondrocytes were exposed to BP or IL-1β for 24 h. Gene expression analysis was performed for *p16* or *p21* (A: *p16*: *P = 0.051 1. B: *p21*: *P = 0.022 9. C: *p16*: **P = 0.009 6. D: *p21* **P = 0.003 9). Bars represent mean ± SEM for *n* = 5 independent experiments**. e** Adeno-GFP or adeno-cre were transduced into IKK2^−/−^ chondrocytes. Cells were then treated with BP and/or IL-1β for 24 h. After 24 h, protein lysates were collected and immunoblotting was performed for p16-Ink4a. Representative immunoblot image is shown. **f** Medial and lateral knee articular cartilage was isolated from patients undergoing TKA with medial compartment OA. P16 gene expression was measured by qPCR (*P* = 0.053 0). Bars are mean ± SD for *n* = 9 patients. **g** Medial and lateral knee articular cartilage was isolated from patients undergoing TKA with medial compartment OA. Cartilage tissue was sectioned and IHC was carried out for p16-Ink4a. Image is representative of *n* = 4 healthy and diseases cartilage sections. **h** GFP or IKK2ca were retrovirally transduced into chondrocytes. p16 expression was measured by qPCR (*P = 0.012 2). Bars are mean ± SD from *n* = 4 independent experiments. **i** IKK2ca was expressed in chondrocytes of adult mice in vivo under the control of the tamoxifen inducible aggrecan-cre (Acan^IKK2ca^). Six weeks post induction, mice were sacrificed and joints were embedded in paraffin and sectioned. IHC was performed for p16, with dark stain representing p16 positive cells. Representative images are displayed. **j**, **k** IKK2^−/−^ chondrocytes were transduced with adeno-GFP or adeno-cre. Cells were then treated with BP for 24 h and *p16* and *p21* gene expression was measured (p16: *P = 0.021 2, *n* = 2. p21: **P = 0.001 7, *n* = 4). Bars represent mean ± SD from representative experiment

Next, we examined if inflammatory stimuli were responsible for promoting cellular senescence. BP or IL-1β treatment increased gene expression of cell cycle inhibitors p16 and p21, which are well-established senescence markers (Fig. 5a–d). We focused further on p16 since it has been shown to be important in senescent OA chondrocytes, made evident by the protection of p16-deficient mice against experimental OA development.^42,43^ Gene expression findings were supported by increased protein levels of p16 upon both IL-1β and BP treatment (Fig. 5e). We also observed increased gene expression of *P16* in damaged compared to undamaged human OA cartilage (Fig. 5f), and increased staining for p16 in OA cartilage (medial condyle) compared to healthy cartilage (lateral condyle) (Fig. 5g). In addition, we show that IL-1β and BP promote the expression of SASP genes Il-6, Lcn2, and Mmp13 (Supplementary Fig. S3a–f). Furthermore, we show that treatment with a well-established senolytic combination of dasatinib and quercetin (D + Q)^44^ was able to significantly reduce expression of SASP factors Il-6, Mmp13, and Lcn2 induced by IL-1β or BP treatment (Supplementary Fig. S3a–f). D + Q combination was also able to partially reverse the decreased expression of aggrecan caused by inflammatory stimuli (Supplementary Fig. S3g). Primers used for all genes are listed in Table 2.

**Table 2:** List of primers used in this study

Gene name	Primer sequence
Actin (mouse)	F- CTAAGGCCAACCGTGAAAAG
	R- ACCAGAGGCATACAGGGACA
IL-6 (mouse)	F- GAGGATACCACTCCCAACAGACC
	R- AAGTGCATCATCGTTGTTCATACA
MMP13 (mouse)	F- GCCAGAACTTCCCAACCAT
	R- TCAGAGCCCAGAATTTTCTCC
IL-6 (human)	F- CCAGCTATGAACTCCTTCTC
	R- GCTTGTTCCTCACATCTCTC
MMP13 (human)	F- AATATCTGAACTGGGTCTTCCAAAA
	R- CAGACCTGGTTTCCTGAGAACAG
NFKBIZ (mouse)	F- TCTCACTTCGTGACATCACC
	R- GGTTGGTATTTCTGAGGTGGAG
NFKBIZ (human)	F-CCGTTTCCCTGAACACAGTT
	R- AGAAAAGACCTGCCCTCCAT
Rankl (mouse)	F- TGAAGACACACTACCTGACTCCTG
	R- CCCACAATGTGTTGCAGTTC
Rankl (human)	F- CACTATTAATGCCACCGAC
	R- GGGTATGAGAACTTGGGATT
OPG (mouse)	F- GTTTCCCGAGGACCACAAT
	R- CCATTCAATGATGTCCAGGAG
P16 (mouse)	F- AATCTCCGCGAGGAAAGC
	R- GTCTGCAGCGGACTCCAT
P16 (human)	F- CCCAACGCACCGAATAGTTA
	R- ACCAGCGTGTCCAGGAAG
P21 (mouse)	F- TTGCCAGCAGAATAAAAGGTG
	R- TTTGCTCCTGTGCGGAAC
P21 (human)	F- TGGAGACTCTCAGGGTCGAAA
	R- GGCGTTTGGAGTGGTAGAAATC
Lcn2 (mouse)	F- CCATCTATGAGCTACAAGAGAACAAT
	R- TCTGATCCAGTAGCGACAGC
Acan (mouse)	F- CCAGCCTACACCCCAGTG
	R- GAGGGTGGGAAGCCATGT
Nlrp3 (mouse)	F- CCCTTGGAGACACAGGACTC
	R- GAGGCTGCAGTTGTCTAATTCC
TRAP (mouse)	F- CACTCCCACCCTGAGATTTGT
	R- CATCGTCTGCACGGTTCTG
Adamts4 (mouse)	F- CTTCCTGGACAATGGTTATGG
	R- GAAAAGTCGCTGGTAGATGGA
Nqo1 (mouse)	F- TTTAGGGTCGTCTTGGCAAC
	R- GTCTTCTCTGAATGGGCCAG
Ho1 (mouse)	F- GTCAAGCACAGGGTGACAGA
	R- ATCACCTGCAGCTCCTCAAA
iNos (mouse)	F- TGCATGGACCAGTATAAGGCAAGC
	R- GCTTCTGGTCGATGTCATGAGCAA
NLRP3 (human)	F- CTTCTCTGATGAGGCCCAAG
	R- GCAGCAAACTGGAAAGGAAG

Suspecting that the senescent shift was occurring due to NF-κB activity, we show that chondrocytes expressing IKK2ca also had increased expression of *p16* (Fig. 5h). In addition, mice expressing IKK2ca in chondrocytes (*Acan*^*IKK2ca*^) display increased p16 expression in the articular cartilage compared to wild type mice (Fig. 5i). Validating these findings, inhibition of NF-κB through deletion of IKK2 was able to decrease the expression of *p16* and *p21* in response to BP stimulation (Fig. 5j, k). This effect was also seen at the protein level, where deletion of IKK2 almost completely inhibited expression of p16 (Fig. 5e). This suggests that NF-κB activity is at least partially a driver of p16-mediated senescent changes in addition to acting as a driver of SASP gene expression.

**Fig. 6:**
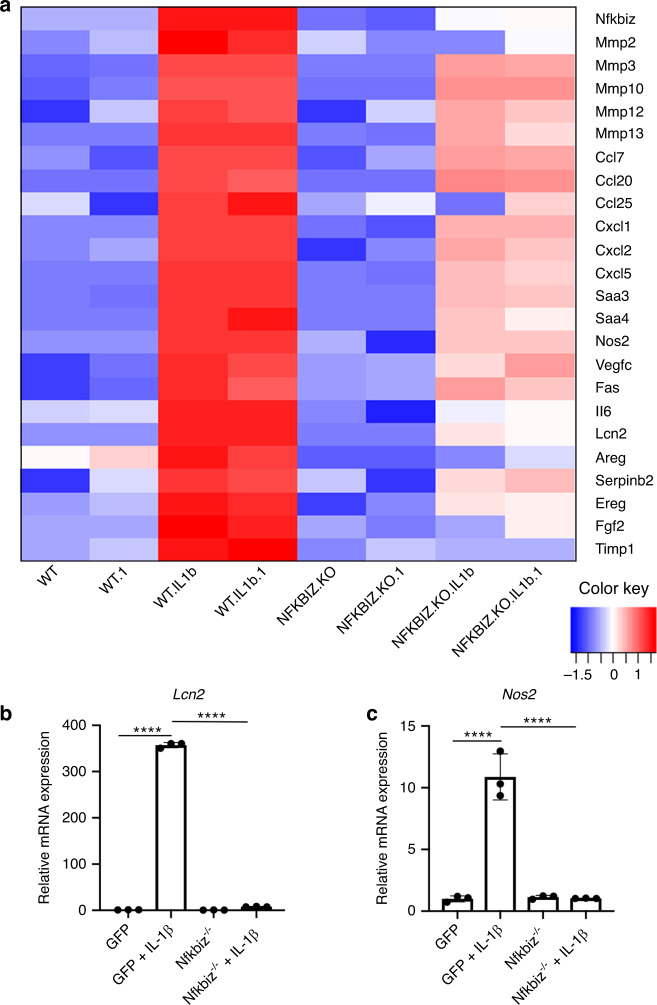
IκB-ζ is a major driver of SASP expression in chondrocytes subject to inflammation. **a** Wild type or *Nfkbiz*^*−/−*^chondrocytes were treated with IL-1β (10 ng·mL^−1^) for 24 h. RNA sequencing was performed and significantly downregulated SASP genes with *Nfkbiz* deletion are displayed. All samples were performed with biological duplicates. **b**, **c** Wild type or *Nfkbiz*^*−/−*^ chondrocytes were exposed to IL-1β (10 ng·mL^−1^) for 24 h. Gene expression of *Lcn2* and *Nos2* was measured by qPCR. Bars are mean ± SD for three samples (****P < 0.000 1)

Since deletion of NF-κB is often detrimental to cell survival, we compared the transcriptome of IL-1β-treated IκB-ζ deficient (*Nfkbiz*^−/−^) chondrocytes to IL-1β-treated wild type chondrocytes^34^ and observed that IκB-ζ deletion decreased expression of many SASP factors associated with senescent chondrocytes (Fig. 6a). We then validated these results by qPCR for SASP genes such as *Lcn2* and *Nos2* to display that IκB-ζ is the principal mediator of inflammation-induced chondrocyte SASP expression downstream of NF-κB (Fig. 6b, c). However, IκB-ζ deletion did not modulate expression of senescence markers, such as *p16* and *p21* (Supplementary Fig. S3h, i). Taken together, our data display that IκB-ζ is the central inflammatory mediator responsible for SASP, while NF-κB separately regulates both SASP and senescent marker changes.

### Inflammatory stimuli promote oxidative stress in chondrocytes to express IκB-ζ and SASP

**Fig. 7:**
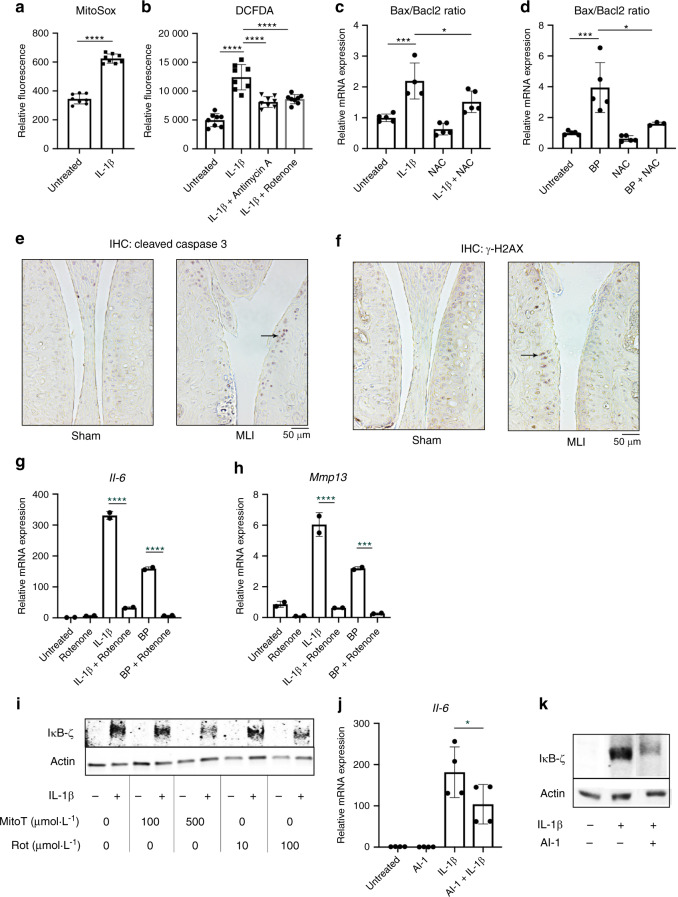
Inflammatory mediators promote apoptosis via mitrochondrial oxidative stress. **a** Mitochondrial superoxide production was measured by MitoSox fluorescence under similar conditions (****P < 0.000 1). Bars are mean ± S. for *n* = 8 replicates from representative experiment. **b** Chondrocytes treated with IL-1β ± antimycin A (10 μmol·L^−1^) or rotenone (100 μmol·L^−1^) for 24 h. ROS levels in the cell were measured by DCFDA fluorescence (****P < 0.000 1). Bars represent mean ± SD for *n* = 8 replicates from representative experiments. **c**, **d** Chondrocytes treated with IL-1β or BP ± NAC (3 mmol·L^−1^) for 24 h. *Bax* and *Bcl2* gene expression normalized to actin expression. *Bax* and *Bcl2* ratios were then determined, normalizing to untreated cells (A: Untreated vs IL-1β ***P = 0.000 5, IL-1β vs IL-1β + NAC *P = 0.041 9, B: Untreated vs BP ***P = 0.000 4, BP vs BP + NAC *P = 0.011 8). Bars are mean ± S.E.M from *n* = 5 independent experiments. **e**, **f** MLI surgery was performed on 12-week-old mice. Control sham surgery performed on contralateral knee. Joints were collected, embedded in paraffin and sectioned. IHC for cleaved caspase-3 and γ-H2AX was performed, with brown stain indicating positive cells. Representative images are displayed. **g, h** Chondrocytes were exposed to IL-1β or BP for 24 h ± rotenone (100 μmol·L^−1^). *Il6* and *Mmp13* gene expression was measured by qPCR (****P < 0.000 1). Bars are mean ± SD from representative experiment. **i** Chondrocytes were treated with IL-1β in the presence or absence of mitoTEMPO or Rotenone doses indicated. Western blotting was performed for IκB-ζ with actin used as control. Representative immunoblot is displayed. **j** Chondrocytes were exposed to IL-1β ± AI-1 (40 μmol·L^−1^) for 24 h. *Il6* gene expression was measured by qPCR. Bars represent mean ± SD from *n* = 4 replicates. **k** Western blot was performed for IκB-ζ under the same conditions. Representative immunoblot is displayed

We then sought to identify a therapeutic target that was driving the expression of IκB-ζ. We have previously shown that IκB-ζ is redox sensitive, with increased cellular ROS causing higher protein expression of IκB-ζ^34^ and sought to determine if IL-1β and BP were drivers of oxidative stress. NOX enzymes are a major cause of reactive oxygen species (ROS) upon inflammatory stimulation of chondrocytes,^45,46^ but unlikely to be the only source. Mitochondria are major ROS producers,^47^ especially when there is mitochondrial dysfunction, and previous studies have shown that chondrocytes treated with inflammatory stimuli display defective mitochondria.^34,48^ For the initial experiments using fluorescent readouts of redox states, we primarily utilized IL-1β since BP interfere with fluorescent assays. We show that chondrocytes treated with IL-1β have increased mitochondrial superoxide production (Fig. 7a), likely from electron transport chain activity (ETC). This is further supported by increased membrane permeability from proteins such as PUMA (Supplementary Fig. S4a), which promotes mitochondrial membrane permeability through the stabilization of Bax.^49^ Together, these changes can lead to ETC dysfunction. Supporting this paradigm, antimycin A and rotenone, both ETC inhibitors, were able to reduce overall cellular ROS levels that were elevated in response to inflammation (Fig. 7b). Furthermore, previous studies have displayed that high oxidative stress can contribute to cell apoptosis as well. We utilized the Bax/Bacl2 ratio as an apoptotic rheostat^50,51^ and show that IL-1β and BP both pushed the cell towards an apoptotic state (Fig. 7c, d). However, the use of NAC, an antioxidant, was able to protect the cell against an apoptotic shift. We then identified in vivo that MLI joints had higher expression levels of cleaved caspase-3, which is an apoptosis marker, as well as increased staining for γ-H2AX, which is a marker of DNA damage that can be associated with oxidative stress, suggesting that these in vitro findings likely extend in vivo as well (Fig. 7e, f).

Given that IL-1β treatment clearly caused mitochondrial dysfunction leading to oxidative stress, we wanted to determine if ETC inhibitors were effective at blocking the IL-1β and BP-induced inflammatory response by reducing oxidative stress. This would validate our findings of inflammation-induced oxidative stress by BP even if BP interfered with fluorescent assays. Mitochondrial ETC inhibitors antimycin A and rotenone treatment reduced expression of IκB-ζ protein and *Il-6* and *Mmp13* inflammatory gene expression (Fig. 7g, h, Supplementary Fig. S4b, c) induced by both IL-1β and BP, likely by decreasing ROS levels (Fig. 7e) in the cell. Likewise, MitoTEMPO, a mitochondrial ROS scavenger, rotenone, and antimycin A were also able to reduce IκB-ζ protein levels (Fig. 7i, Supplementary Fig. S4d). This corroborates previous findings wherein the use of an ETC inhibitor was able to reduce OA development in an intra-articular fracture model of OA.^52^ Finally, since we suspected oxidative stress to be an important mediator of catabolic gene expression in chondrocytes, we sought to determine if activation of the Nrf2 antioxidant system can reduce catabolic gene expression. We used an Nrf2 pathway activator, AI-1, to display that inflammatory gene expression was reduced with antioxidant gene expression (Fig. 7j). Furthermore, treatment with AI-1 was able to decrease IκB-ζ protein levels (Fig. 7k). AI-1 activity was confirmed by measuring the increased expression of *Ho1* and *Nqo1* (Supplementary Fig. S4e, f). Overall, our data demonstrate that NF-κB and IκB-ζ mediated inflammatory and catabolic responses in chondrocytes are regulated by mitochondria-dependent oxidative stress.

### Nitrosative stress can promote IκB-ζ expression but may not be pro-inflammatory physiologically

After displaying the role of mitochondrial dysfunction as a source of oxidative stress, we then wanted to explore the role of nitrosative species such as nitric oxide (NO), which can combine with ROS to form new damaging molecules.^53^ Prior studies have suggested NO is a pathological species in OA but its relation to chondrocyte senescence and inflammatory response is not well understood.

**Fig. 8:**
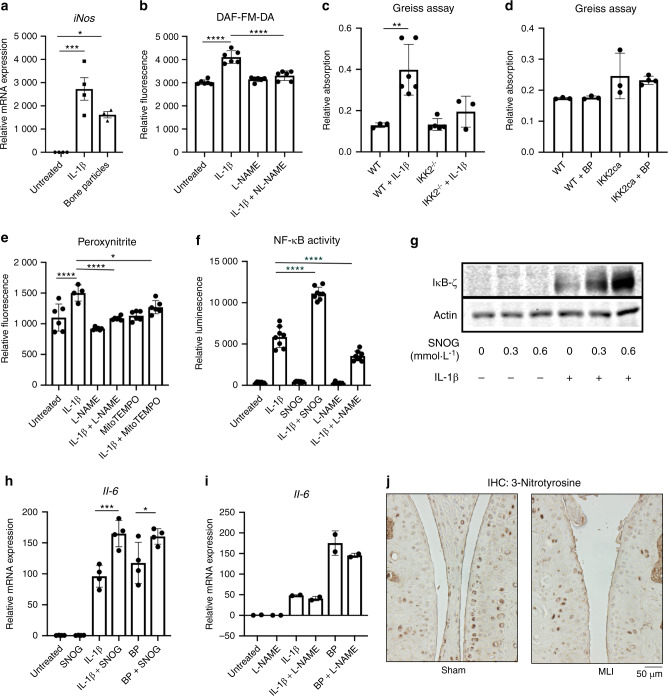
Nitrosative stress has different effects on inflammatory response in vitro and in vivo. **a** Chondrocytes were treated with IL-1β or BP for 24 h. Gene expression of *iNos* was measured by qPCR (F: Untreated vs IL-1β ***P = 0.000 6, Untreated vs BP *P = 0.020 1). **b** Chondrocytes were treated with IL-1β ± L-NAME (75 μmol·L^−1^) for 24 h. NO levels were measured using DAF-FM-DA fluorescent probe. Bars represent mean ± SD of *n* = 8 replicates from representative experiment (****P < 0.000 1). **c** WT or IKK2^−/−^ chondrocytes were treated with IL-1β for 24 h. Supernatant was collected and Greiss assay was carried out to measure NO production. (WT vs WT + IL-1β **P = 0.002 6). Results are representative of at least 3 biological replicates. **d** Chondrocytes were transduced with pMX-GFP (WT) or pMX-IKK2ca. They were then treated with BP for 24 h. Supernatant was collected and NO production was performed using Greiss assay. Results are representative of at least three biological replicates. **e** Chondrocytes treated with IL-1β ± L-NAME (75 μmol·L^−1^) or MitoTEMPO (0.5 mmol·L^−1^) for 24 h. Peroxynitrite levels were measured using fluorescent sensor dye (Untreated vs IL-1β ****P < 0.000 1, IL-1β vs IL-1β + L-NAME ****P < 0.000 1, IL-1β vs IL-1β + MitoTEMPO *P = 0.045). Bars are mean ± SD from *n* = 4-6 replicates. **f** NF-κB luciferase reporter chondrocytes treated with IL-1β ± SNOG or L-NAME for 24 h. Luciferase activity was measured as a readout of NF-κB activation (****P < 0.000 1). Bars are mean ± SD for *n* = 8 replicates from representative experiments. **g** Primary chondrocytes treated with IL-1*β* ± S-Nitrosoglutathione (0.6 mmol·L^−1^) for 24 h. Western blot was performed for IκB-ζ. Representative immunoblot is displayed. **h** Chondrocytes were treated with IL-1β or BP ± SNOG (0.6 mmol·L^−1^). *Il6* gene expression measured by qPCR (B: IL-1β vs IL-1β + SNOG ***P = 0.000 6, BP vs BP + SNOG *P = 0.04). Bars are mean ± SD of *n* = 4 independent experiments for Panel (**b**). **i** Chondrocytes treated with IL-1β or BP ± L-NAME (75 μmol·L^−1^). *Il6* gene expression was measured by qPCR. Data represent mean ± SD for replicates from one representative experiment out of three. **j** MLI surgery was performed on 10-week-old mice. Control sham surgery was done on contralateral knee. Joints were collected, embedded in paraffin and sectioned. IHC for 3-nitrotyrosine was performed, with brown stain indicating positive cells. Representative images are displayed

We first show that IL-1β and BP treatment increased iNOS expression (Fig. 8a), an intracellular enzyme responsible for NO production. We also display that IL-1β increased NO levels in these cells (Fig. 8b), as measured with a fluorescent probe. BP technically interfered with the fluorescent assay used but were predicted to cause an increase in NO based on the elevated iNOS expression. However, using the Greiss assay to measure NO secretion from chondrocytes in vitro, we were able to show that IL-1β significantly increased NO production (Fig. 8c) but BP were unable to drive chondrocyte NO production (Fig. 8d), suggesting a mechanistic difference between BP and IL-1β in mediating NO production. Furthermore, we displayed that the effect observed with IL-1β was dependent upon the IKK2-NF-κB axis, since IKK2^−/−^ chondrocytes had significantly lower NO production (Fig. 8c). To confirm that the elevated NO is originating from iNOS, we used L-NAME, a known iNOS inhibitor, to show that it is able to block the IL-1β mediated increase in NO (Fig. 8b).

NO can pathologically combine with superoxide from the mitochondria to form peroxynitrite, which is a known pathogenic ROS.^53^ Using a peroxynitrite-specific fluorescent probe, we display that peroxynitrite levels increase with IL-1β treatment in chondrocytes (Fig. 8e). Peroxynitrite reacts with proteins to form 3-nitrotyrosine (3-NT), which is an indicator of oxidative stress and damage that other groups have shown to be elevated in OA cartilage.^54^ We show that peroxynitrite and 3-NT levels elevated by IL-1β decreased with L-NAME treatment (Fig. 8e, Supplementary Fig. S5a), indicating that the NO arising from iNOS contributes to damaging peroxynitrite formation. Furthermore, since we suspected that NO combines with superoxide from the mitochondria, we show that mitoTEMPO, a mitochondria-targeted antioxidant, is able to decrease peroxynitrite formation (Fig. 8e).

After observing that oxidative stress can drive senescence and SASP formation in an NF-κB-IκB-ζ mediated manner, we sought to interrogate the role of NO in this system. We treated chondrocytes with an NO donor, S-nitrosoglutathione (SNOG), which we confirmed was able to increase NO levels and 3-NT formation (Supplementary Fig. S5b, c). We observed that SNOG was pro-inflammatory, increasing NF-κB activation by IL-1β as well as protein levels of IκB-ζ (Fig. 8g, h). Furthermore, it was able to promote IL-1β and BP-induced expression of IL-6 (Fig. 8i). However, the use of iNOS inhibitor, L-NAME, was unable to decreased IL-1β and BP-induced expression of IL-6, suggesting that intrinsic NO levels are unlikely to be pro-inflammatory (Fig. 8j).

Since we had shown that oxidative stress promotes senescence and apoptosis, we wanted to determine if NO had similar pathologic effects. SNOG treatment in the setting of inflammatory stimuli leads to a significant increase in p21 expression, and a slight, although non-significant increase in p16 expression (Supplementary Fig. S5d, e). There was also a slight increase in Bax/Bcl2 ratio with SNOG treatment, though it was not statistically significant (Supplementary Fig. S5f). Furthermore, use of L-NAME, an iNOS inhibitor, was unable to reduce Bax/Bcl2 ratio caused by IL-1β or BP as NAC was able to (Fig. S5g, h), suggesting that a reduction of NO levels alone is insufficient to prevent apoptotic changes since there is still production of damaging oxidative species such as superoxide. Overall, these results suggest that addition of extra-physiological levels of NO with a donor can be pro-inflammatory but intrinsic NO production is not in and of itself pro-inflammatory.

We then sought to determine how these findings translate to the in vivo MLI model. Interestingly, we observed that MLI joints had consistently decreased 3-NT staining in the articular cartilage compared to sham joints, specifically with decreased staining in articular chondrocytes. This finding suggests that contrary to its role in other synovial joint components, 3-NT may not be a reliable marker of inflammation in articular chondrocytes in OA and that intrinsic NO may not be a pro-inflammatory mediator, as we observed in vitro. These results indicate the need for further research into the role of NO in the context of joint inflammation.

## Discussion

This work focuses on understanding the pathogenesis of OA through the lens of inflammation, senescence, and oxidative stress. The presence of mineral crystals and other matrix breakdown products in the synovial fluid has been noted in patients with OA and rheumatoid arthritis.^19,20,22,27,28^ However, it has been difficult to interpret the role of these particles in OA since it is unclear whether they are a product or drivers of the disease. While still unclear, we predict that they may arise from cartilage and bone erosions in part due to osteoclast activity. It is well recognized that patients with late-stage OA have subchondral sclerosis, though some groups have demonstrated that early OA may actually present with decreased subchondral bone volume.^32^ We show that post-traumatic OA (PTOA) joints display increased osteoclast numbers in the subchondral region. We also display that early OA joints have decreased subchondral bone, supporting the finding of increased bone breakdown early in disease, likely by osteoclasts. This leads to a dysregulation in bone remodeling, eventually leading to subchondral sclerosis seen in late-stage OA. Furthermore, we propose that chondrocyte-produced Rankl in response to inflammatory stimuli may be a significant osteoclast promoting force in regions adjacent to damaged cartilage in vivo. It is conceivable that “inflammatory chondrocytes” may recruit OCs and their progenitors to cartilage and adjacent bone matrices by Rankl secretion. Hence, subchondral bone-cartilage interaction is likely to be an important factor in OA disease progression, not simply a by-product of joint damage, as several groups have identified increased osteoclast numbers in subchondral bone,^55–57^ leading to attempts at OA therapy through modulation of subchondral bone remodeling.^58^

The activity of these inflammatory chondrocytes is driven by pathological mediators generated in the joint. Regardless of their source, BP are an example of one such mediator that drive a catabolic response in an NF-κB dependent manner, especially when they synergize with other inflammatory stimuli such as IL-1β. While the BP we utilized are not the same crystals found in the synovial fluid of human OA joints, we suspect that they can have similar pro-inflammatory effects as other inert crystal compounds. Given that other groups have already studied the effect of small crystals on the chondrocyte catabolic response,^20,21,27,28^ we wanted to take an approach that may be more physiologically relevant to disease states in which there is bony erosions or joint injuries leading to BP in the synovial space. We utilized real BP that vary in size and shape, as well as composed of diverse ECM compounds. Some physicians have endorsed the use of synovial lavage for the treatment of OA, although a meta-analysis displayed no benefit over placebo.^59^ However, this technique may be useful in patients with significant calcified compounds in their synovial space early in the disease and should be further studied.

We observed that BP and IL-1β generate a feed forward cycle of inflammation that contributes to the chronic inflammatory state present in OA joints that cannot be targeted by biologics such as anti-IL-1 or anti-TNF therapies, but can be addressed by targeting downstream signaling pathways such as the NF-κB pathway, which integrate signals downstream of several cytokines/receptors. However, given the indispensable role of NF-κB in normal physiologic pathways, sustained blockage of this pathway in humans is counterproductive. Instead, we identified IκB-ζ and NLRP3 inflammasome as downstream mediators of BP-induced inflammation, highlighting these as potential therapeutic targets.

We then provided evidence that inflammatory stimuli can promote senescence via activation of the NF-κB signaling pathway, which promotes various senescent programs such as the p16-Ink4a pathway, and also SASP expression via the NF-κB-IκB-ζ axis. We identified IκB-ζ at the intersection of inflammation and senescence given its ability to drive the expression of pro-inflammatory SASP genes in response to inflammatory stimuli such as BP or IL-1β. We also found that IκB-ζ appeared to be upstream of *Nrlp3* and *Rankl* expression, further asserting its importance in the control of inflammatory, degradative pathways in the joint. Given that the contribution of senescent chondrocytes to OA has been well-established, identifying factors such as IκB-ζ to ameliorate SASP production may be highly beneficial for preventing OA progression. In order to target IκB-ζ, we display that its expression is driven by a combination of inflammatory stimulation, oxidative and nitrosative stress to promote the expression of SASP genes, providing multiple avenues for reducing SASP expression.

Another interesting finding was that while IL-1β and BP are both pro-inflammatory, they have different effects on ROS and RNS. While IL-1β strongly induces ROS and RNS production, BP appear to be poor inducers of RNS, which may be explained by differences in their signaling pathways. Both induce NF-κB activation to drive inflammation, but they may have unique alternative pathway activation regarding nitrosative stressors. We identified the mitochondria as one source of oxidative stress activated by both players that appears to be a key driver of the inflammatory response through superoxide production. We further found that ETC inhibitors may be effective for treatment in the context of inflammation by blocking IκB-ζ expression, but that iNOS inhibitors were not effective. Hence, the role of RNS in the context of disease pathology requires further elucidation, especially in vivo, where we unexpectedly saw decreased 3-NT staining in the articular chondrocytes of OA joints. It is likely that NO can have pro-inflammatory functions at supraphysiological levels, which in conjunction with ROS species is likely to form molecules such as peroxynitrite, consistent with our finding that NO donors can exacerbate an inflammatory response in vitro by stabilizing IκB-ζ. This would corroborate some studies that have suggested that 3-NT levels are a pathological marker that is elevated in OA.^52,58^ However, we observed the opposite results in the chondrocytes in our animal model, which may be a function of the MLI mouse model of OA. Given that most of our work focuses on chondrocytes, it will be important to further understand the role of NO and 3-NT in other joint components such as synoviocytes, synovial macrophages and subchondral bone, which are intricately related to the articular cartilage, and may reconcile our work with prior studies displaying elevated 3-NT in OA joint synovial fluid, synovial cells and tissues. The lack of protection with iNOS inhibition in vitro further indicates that ROS species such as superoxide may be the primary inflammatory mediators and NO by itself is less important for chondrocyte inflammation. Another explanation is that exogenous NO donors produce NO throughout the cell, while physiologically, there are unique isoforms of iNOS that exist within subcellular compartments such as mitrochondrial NOS, iNOS, eNOS, and nNOS.^60^ Further supporting our findings that NO may not be pathologic, other groups have also displayed that NO can actually block mitochondrial ETC activity, leading to an antioxidant and protective effect by reducing superoxide levels.^61,62^ Given these findings, it is likely that NO has unique spatial and temporal effects within chondrocytes and needs to be better understood in the context of the OA joint through deletion of specific NOS subtypes and better NO detectors.

There are several experiments needed to further build upon the foundation of this work. First, we seek to develop a chondrocyte-specific Rankl knockout mouse to characterize the role of Rankl in OA progression and better appreciate the relationship between articular cartilage and subchondral bone, an area in need of significant exploration. A shortcoming of this project was the use of primarily murine chondrocytes, though we did utilize human OA cartilage for studying gene expression in order to validate our findings. The use of human synovial fluid to measure BP and the factors present in the fluid can provide insight into the role of these molecules in human disease. Another limitation of this project was the performance of in vitro experiments at 21% oxygen, which can influence the understanding of oxidative and nitrosative pathways, though we have displayed in a previous publication that inflammatory responses are similar at 21% oxygen and 4% oxygen cultures.^34^ Finally, it is important to use large animal models to validate our findings regarding oxidative stressors in the joint. The thickness of joint cartilage varies significantly from species to species, which can affect the ability of oxidative species to travel and affect adjacent cellular compartments. The use of pig and bovine models to validate our results will provide greater insight into the role of subchondral remodeling as well as oxidative stress in the joint.

## Materials and methods

### Animal models

All animal models were bred on a C57/BL6 background. 10-week-old wild type mice were used for PTOA models. For isolation of primary chondrocytes, WT, *IKK2*^*f/f*^, *Nfkbiz*^f/f^, and Nlrp3 KO pups aged P1-P3 were used. Recombination was induced in Aggrecan-ERT2-cre mouse model by feeding tamoxifen chow for 2 weeks. IKK2ca flox/flox mice were crossed with Aggrecan-ERT2-cre mice to express IKK2ca in chondrocytes in mature mice at age 10 weeks. All experiments were performed using littermate controls. Mice were housed at the Washington University School of Medicine barrier facility. All experimental protocols were carried out in accordance with the ethical guidelines approved by the Washington University School of Medicine Institutional Animal Care and Use Committee. Mice were housed in barrier facility at five or less per cage at 24–26 degrees Celsius with humidity ranging between 30%–60% with 12 h light/dark cycles switching at 6 pm.

### Meniscal ligamentous injury model

The MLI model was utilized to induce post-traumatic OA in mice at 12 weeks of age. Medial ligament and meniscus were transected in the right legs of mice, with sham surgery performed on the contralateral knee joint. Sham surgery involves cutting skin superficially and suturing without transecting ligaments in the knee joint. Mice were sacrificed and limbs were harvested for further analysis.

### Cell culture

Primary chondrocytes were harvested from sterna and ribs of P1-P3 mice using sequential digestion with pronase (2 mg·mL^−1^, PRON-RO, Roche) at 37 degrees, collagenase D (3 mg·mL^−1^, COLLD-RO, Roche) two times at 37 degrees, and cultured in DMEM (Life Technologies, USA) containing 10% FBS and 1% penicillin/streptomycin (Thermo Fisher). Primary chondrocytes are not passaged and experiments are completed within 5 days of isolation from mice. Primary macrophages (osteoclast precursors) were harvested from long bones of WT mice and cultured in M-CSF overnight before being added to chondrocyte cultures. Cells were incubated with recombinant IL-1β (10 ng·mL^−1^), IKK2 inhibitor SC-514 (10 μmol·L^−1^), BP (1 mg·mL^−1^). Plat-E cells were used to generate retroviral particles.

### Bone particle generation

BP were obtained from porcine long bones. First, porcine bones were obtained from butcher shops. Then bone surfaces were extensively washed with PBS and 70% ethanol. The cortical bone was then cut using an automatic low speed bone saw (Buehler IsoMet) on the epiphyseal surfaces. The bone dust produced was collected as BP. The BP were then thoroughly washed with sterile PBS, autoclaved and stored in 70% ethanol at 4 degrees. Prior the use, BP were washed in sterile PBS to remove ethanol and re-suspended in PBS. After shaking tube containing particles, large orifice pipette tips were used to pick up BP from the surface of the solution and added to chondrocyte cell culture.

### Retroviral and adenoviral infection

Adenovirus was utilized for deleting genes from cells with floxed genes. Cells were infected with commercially obtained adenoviral-GFP (VVC-U of Iowa 4) and adenoviral-cre (VVC-U of Iowa 3554) at an MOI of 10 in the presence of 5 μg·mL^−1^ of polybrene (TR-1003, Sigma) in 5% FBS-containing-DMEM. After 24 h, media was switched to DMEM + 10% FBS. Retrovirus was utilized to infect chondrocytes with GFP, IKK2ca, or IKK2-KD. Retrovirus was generated by transfecting PLAT-E cells with pMX-GFP, pMX-IKK2ca or pMX-IKK2-KD constructs using X-tremegene 9(XTG9-RO, Roche) and media was changed after 24 h. After 48 h, supernatant containing retrovirus was used to infect primary chondrocytes in the presence of 5 μg·mL^−1^ of polybrene. After 24 h of infection, media was changed and chondrocytes were treated as stated.

### Western blotting

Cell lysates were collected in 1x cell lysis buffer (Cell Signaling Technology, Danvers, MA, USA) with protease/phosphatase inhibitor (Thermo Fisher Scientific). Samples were denatured in 1x sample buffer containing β-ME by boiling for 10 min. Samples were analyzed using PAGE electrophoresis. Membranes were incubated with desired primary and secondary antibodies. Signals were captured using LiCor Odyssey reader (LI-COR Biosciences, Lincoln, NE, USA). Primary antibodies used were anti-IκB-ζ (Cat# 14-16801-82, Invitrogen, 1:1 000), anti-p16, anti-PUMA and anti-Actin (Cat# A228, Sigma, 1:5 000 dilution).

### Quantitative PCR

Trizol (Sigma) and Chloroform at a ratio of 0.2:1 were added to samples, followed by centrifugation at 12 000 × g for 15 min. Aqueous layer was collected and equal amount of 70% ethanol was added. RNA was then isolated from this fraction using PureLink RNA mini kit (Cat# 12183025, Ambion, Grand Island, NY, USA). cDNA was prepared using High Capacity cDNA Reverse Transcription kit (Cat# 4368814, Applied Biosystems). qPCR was carried out on BioRad CFX96 real time system using iTaq universal SYBR green super-mix (Cat#1725120, BioRad, Hercules, CA, USA). Actin was used to normalize mRNA expression.

### IL-1β ELISA

Chondrocytes were cultured with BP and/or IL-1β. Supernatant was collected for ELISA. Supernatant was added to IL-1β-coated ELISA plates and incubated overnight at 4 degrees. Samples were washed with PBST and biotin-conjugated detection antibody was added to plates for 1 h at room temperature. After washing again, streptavidin-HRP was added to wells for 1 h, washed, and TMB substrate was added. Reaction was stopped prior to reading.

### Histology

Knee joints were isolated from mice and fixed in 10% neutral buffered formalin (HT501128, Sigma) for 24 h followed by decalcification in Immunocal (StatLab, McKinney, TX) for 3 days. Samples were then embedded in paraffin before cutting 5 μmol·L^−1^ sections. Unstained sections were utilized for IHC. Remaining sections were stained with safranin-O/fast green stain. Severity of OA was graded by OARSI scoring. Reviewers were blinded to treatment conditions of slides being scores.

### Immunohistochemistry

Sections were deparaffinized and rehydrated using three changes of xylenes followed by ethanol gradient. Antigen retrieval was performed using citrate solution pH 6.0 at 60 ℃ overnight, followed by blocking for 1 h using blocking solution (10% goat serum in PBS, 1% tween-20, 1% BSA). Sections were then incubated with primary antibody diluted in DAKO dilution solution (S3022, Agilent, Santa Clara, CA) overnight at 4 degrees then washed and incubated with biotin-conjugated secondary antibody (BP-1100, Vector Biolabs, Burlingham, CA) at room temperature for 2 h. Sections were washed and incubated with streptavidin-HRP using Vectastain ABC-HRP (PK-4000, Vector Laboratories, Burlington, CA) for 20 min. After washing, sections were developed using DAB peroxidase kit (SK4100, Vector Laboratories, Burlingham, CA). Anti-p16-ink4a (PA5-20379, Thermo Fisher), anti-IκB-ζ (NBP-89835, Novus Biologicals, Centennial, CO), anti-3-NT (06-284, Millipore Sigma, Burlington, MA), cleaved caspase-3 (9661 S, CST, Danvers, MA), and Υ-H2AX (2577 S, CST, Danvers, MA) were used in blocking solution (1:100 dilution).

### ROS and RNS assays

ROS species in the cell were measured using H2, DCF-DA fluorescent dye (Cat#D6883, Sigma) and RNS species were measured using H2-DAF-FM-DA fluorescent dye in 96 well plate format. Cells were pre-treated 24 h with appropriate treatment conditions in DMEM media. Next, cells were washed and incubated with 5 μmol·L^−1^ DCFDA or DAFFM-DA dissolved in PBS for 30 min, washed several times with PBS and placed back in the incubator. After 1 h, fluorescence was measured on microplate reader at Ex/Em 495/518.

### Peroxynitrite assay

Peroxynitrite was measured in the cell using Dax-J2 PON fluorescent dye (Abcam ab233469) in 96 well plate format. Cells were pre-treated for 24 h with appropriate treatment conditions in DMEM media. Cells were incubated with 10 μmol·L^−1^ Dax-J2 PON in media for 30 min. Next, cells were washed several times with PBS and placed back in the incubator. After 1 h, fluorescence was measured on microplate reader at Ex/Em 495/518.

### 3-Nitrotyrosine ELISA

3-Nitrotyrosine adduct formation on proteins in the cell were measured by ELISA kit (Abcam, ab116691tab). Cells were treated with appropriate conditions for 24 h in six well plates. ELISA was then performed according to protocol for the assay with no modifications.

### Rankl ELISA

Rankl secretion by chondrocytes in vitro was measured by ELISA kit (Abcam, Ab100749). Chondrocytes were cultured with IL-1β for 24 h and 100 μL of supernatant was used undiluted in the assay, performed in technical replicates for each biological sample.

### Greiss assay

Supernatant from chondrocyte culture was collected under various conditions. Greiss assay was performed using assay kit (G7921, Thermo Fisher) in microplate format, using 150 uL of supernatant per well. Culture media was used as control for background correction.

### Micro computed tomography (μCT)

Intact knee joints were harvested, fixed overnight in 10% neutral buffered formalin (HT501128, Sigma) then washing with Phosphate Buffer Saline (PBS) three times and transfered to 70% ethanol (v/v). Bones were scanned at a resolution of 20 µm, slice increment 10 µm, voltage 55 kV, current 145 µA and exposure time of 200 ms and 3D images were constructed using Scanco Medical micro-CT systems (Scanco, Wayne, PA, USA) at Washington University in St. Louis (St. Louis, MO).

### RNA sequencing

Primary chondrocytes were cultured ± IL-1β (10 ng·mL^−1^) for 24 h. RNA was collected and samples were prepared according to library kit manufacturer’s protocol, indexed, pooled, and sequenced on an Illumina HiSeq. Basecalls and demultiplexing were performed with Illumina’s bcl2fastq software and a custom python demultiplexing program with a maximum of one mismatch in the indexing read. RNA-Seq reads were then aligned to the Ensembl release 76 primary assembly with STAR version 2.5.1a. Gene counts were derived from the number of uniquely aligned unambiguous reads by Subread:featureCount version 1.4.6-p5. Isoform expression of known Ensembl transcripts were estimated with Salmon version 0.8.2. Sequencing performance was assessed for the total number of aligned reads, the total number of uniquely aligned reads, and features detected. The ribosomal fraction, known junction saturation, and read distribution over known gene models were quantified with RSeQC version 2.6.2.

All gene counts were then imported into the R/Bioconductor package EdgeR and TMM normalization size factors were calculated to adjust for samples for differences in library size. Ribosomal genes and genes not expressed in the smallest group size minus one sample greater than one count-per-million were excluded from further analysis. The TMM size factors and the matrix of counts were then imported into the R/Bioconductor package Limma. Weighted likelihoods based on the observed mean-variance relationship of every gene and sample were then calculated for all samples with the voomWithQualityWeights. The performance of all genes was assessed with plots of the residual standard deviation of every gene to their average log-count with a robustly fitted trend line of the residuals. Differential expression analysis was then performed to analyze for differences between conditions and the results were filtered for only those genes with Benjamini-Hochberg false-discovery rate adjusted *P*-values less than or equal to 0.05.

For each contrast extracted with Limma, global perturbations in known Gene Ontology (GO) terms, MSigDb, and KEGG pathways were detected using the R/Bioconductor package GAGE^8^ to test for changes in expression of the reported log 2-fold-changes reported by Limma in each term versus the background log 2-fold-changes of all genes found outside the respective term. The R/Bioconductor package heatmap3 was used to display heatmaps across groups of samples for each GO or MSigDb term with a Benjamini-Hochberg false-discovery rate adjusted *P*-value less than or equal to 0.05. Perturbed KEGG pathways where the observed log 2-fold-changes of genes within the term were significantly perturbed in a single-direction versus background or in any direction compared to other genes within a given term with *P*-values less than or equal to 0.05 were rendered as annotated KEGG graphs with the R/Bioconductor package Pathview.

### Statistical analysis

Experiments were routinely carried out in replicates, unless otherwise stated. GraphPad Prism was employed for all experiments using appropriate statistical test. Multiple treatments were analyzed by One-way ANOVA followed by Tukey’s test multiple comparisons test. Student’s *T* test was used for comparing two groups. Age and sex-matched mice were used. Values are expressed as mean ± SD of representative experiment out of at least three independent experiments. *P*-values are indicated where applicable.

## Supplementary information

IκB-ζ signaling promotes chondrocyte inflammatory phenotype, senescence, and erosive joint pathology
