# Supplementary material for: IκB-ζ signaling promotes chondrocyte inflammatory phenotype, senescence, and erosive joint pathology
Source: Bone Res. 2022 Feb 11;10:12. doi: 10.1038/s41413-021-00183-9 (PMC8831569; doi:10.1038/s41413-021-00183-9)
Supplement: Supplementary file 1 — IκB-ζ signaling promotes chondrocyte inflammatory phenotype, senescence, and erosive joint pathology [file 41413_2021_183_MOESM1_ESM.docx]

**Supporting information:**


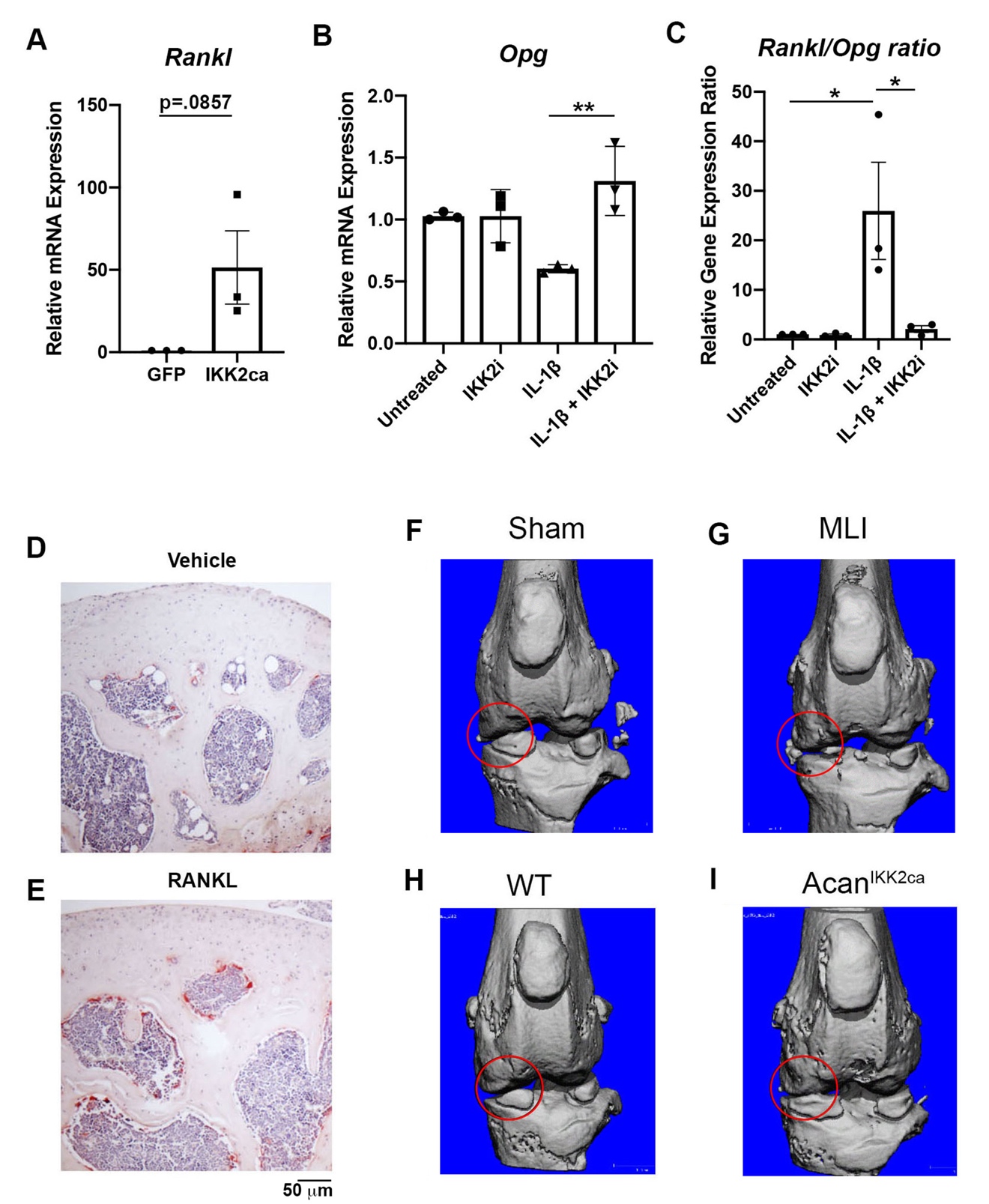


**Supplementary Fig S1**. (A) Primary chondrocytes were retrovirally transduced with GFP or IKK2ca. Gene expression of Rankl was measured by qPCR. Bars represent mean ± S.E.M. from n=3 independent experiments. (B) Primary chondrocytes were treated with IL-1β in the presence or absence of IKK2 inhibitor (10 μM) for 24 hours. Gene expression of *Opg* was measured by qPCR (IL-1β vs IL-1β + IKK2i **P=.0054). Bars represent mean ± S.D. from n=3 independent experiments. (C) *Rankl:Opg* ratio was obtained by diving *Rankl* fold change by *Opg* fold change (Untreated vs IL-1β *P=.0292, IL-1β vs IL-1β + IKK2i *P=.0364). Bars represent mean ± S.D. from n=3 independent experiments. (D-E) Mouse knee joints were injected with PBS or Rankl. After 1 week, knee joints were fixed and sections for TRAP staining. Representative images are displayed. (F-G) MLI surgery was performed on mice, with sham surgery performed on the contralateral limb. μCT was performed to look at erosion of bones surfaces on medial aspect of both limbs (circles), with representative images shown from one mouse. (H-I) Control WT and Aggrecan-ERT2-cre IKK2ca mice were fed tamoxifen for 2 weeks. After 8 weeks, limbs were collected and μCT was performed to look at erosion of bones surfaces (circles) on medial sides of both right lower limbs from representative mice.

**
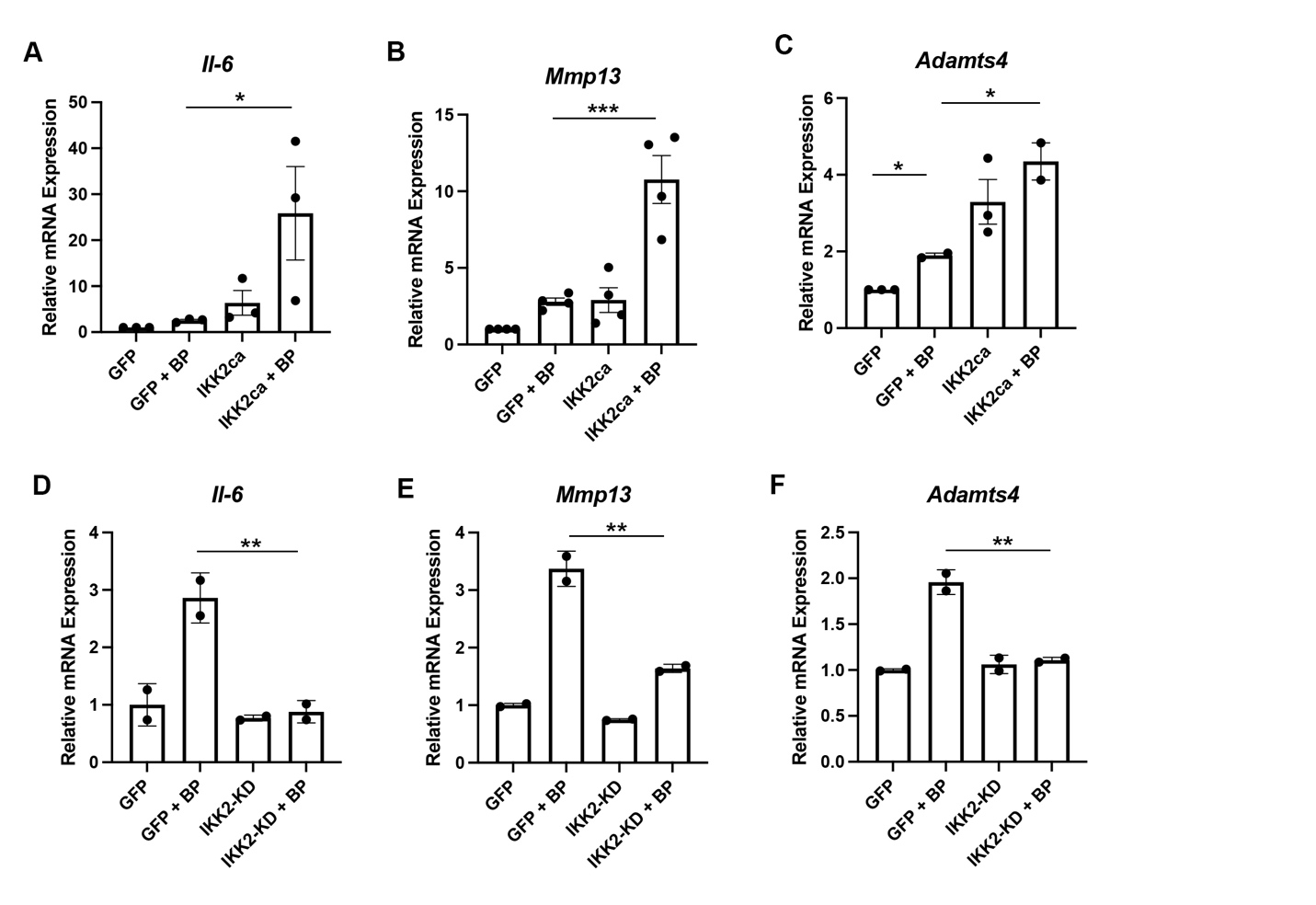
**

**Supplementary Fig S2.** (A-F) Chondrocytes were retrovirally transduced with GFP, IKK2ca or IKK2-KD. Cells were then treated with BP for 24 hours. Gene expression analysis was performed for *Il-6, Mmp13* and *Adamts4* (A: GFP + BP vs IKK2ca + BP *P=0.05, B: GFP + BP vs IKK2ca + BP ***P=.0002, C: GFP + BP vs IKK2ca + BP *P=.0348, D: GFP + BP vs IKK2-KD + BP **P=.0079, E: GFP + BP vs IKK2-KD + BP **P=.0014. F: GFP + BP vs IKK2-KD + BP **P=.002). Results are from one representative experiment out of three performed in duplicates.

**
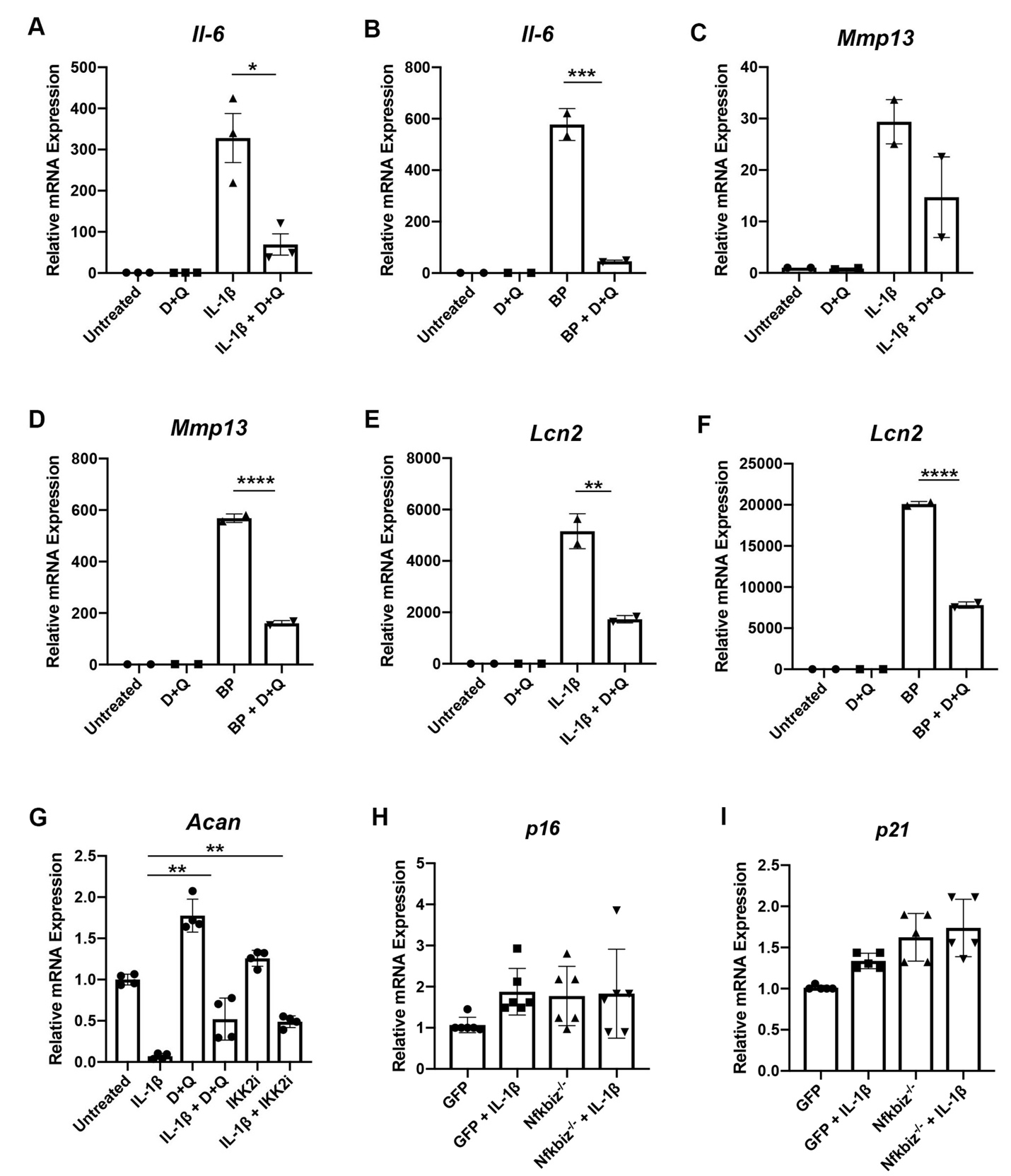
**

**Supplementary Fig S3.** (A-F) Primary chondrocytes were treated with IL-1β or BP in the presence or absence of D+Q (40 nM, 600 μM respectively) for 24 hours. Gene expression of *Il6, Mmp13* and *Lcn2* were measured by qPCR (A: IL-1β vs IL-1β + D+Q *P=.05. B: BP vs BP + D+Q ***P=.0002. D: BP vs BP + D+Q ****P<.0001. E: IL-1β vs IL-1β + D+Q **P=.0021. F: BP vs BP + D+Q ****P<.0001). Bars represent mean ± SEM for independent experiments for Panel A-B. Bars represent mean ± S.D. from one representative experiment for panels C-F. (G) Gene expression of *Acan* was measured by qPCR as well in chondrocytes treated with IL-1β in the presence or absence of IKK2i or D+Q (H: IL-1β vs IL-1β + D+Q **P= .0038, IL-1β vs IL-1β + IKK2i **P=.0074). Bars represent mean ± S.D. for n=4 replicates. (H-I) Wild type or *Nfkbiz^-/-^* chondrocytes were treated with IL-1β (10 ng/mL) for 24 hours. Gene expression of *p16* and *p21* was measured by qPCR. Bars represent mean ± S.D from at least 5 independent experiments (P>.05).


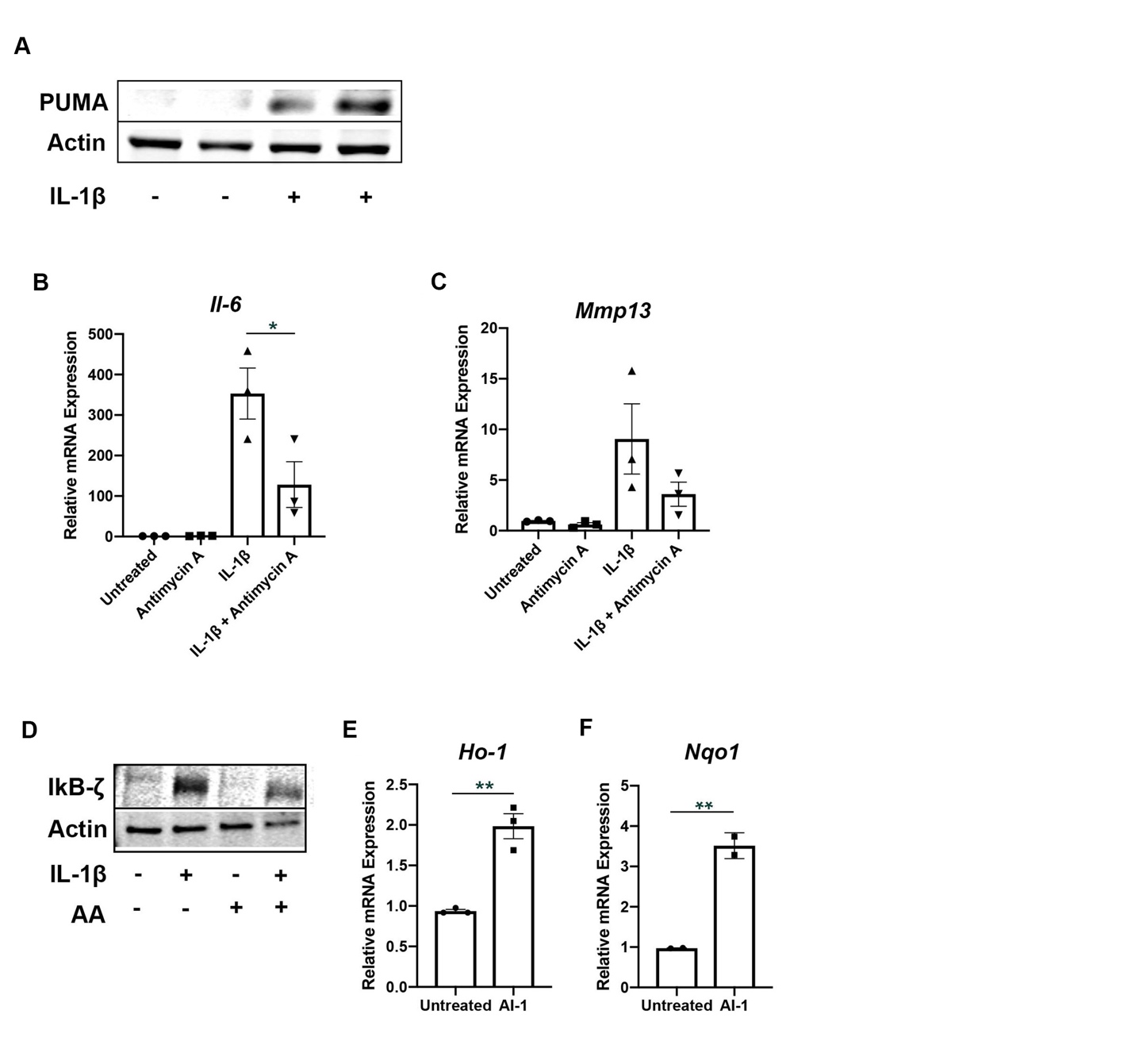


**Supplementary Fig S4.** (A) Chondrocytes were treated with IL-1β for 24 hours in biological duplicates. Immunoblotting was performed for PUMA, with representative immunoblot displayed. (B-C) Chondrocytes were treated with IL-1β in the presence or absence of Antimycin A for 24 hours. Gene expression of *Il6* and *Mmp13* was measured by qPCR (IL-6: *P=.023). Bars represent mean ± SEM for n=3 independent experiments. (D) Western blotting was performed for IκB-ζ under similar conditions. Representative image is displayed. (E-F) Chondrocytes were treated with AI-1 (40 μM) for 24 hours. Gene expression of *Ho1* and *Nqo1* were measured by qPCR. (D: **P= .0026, E: **P=.0079). Bars represent mean ± SEM from independent experiments.

**
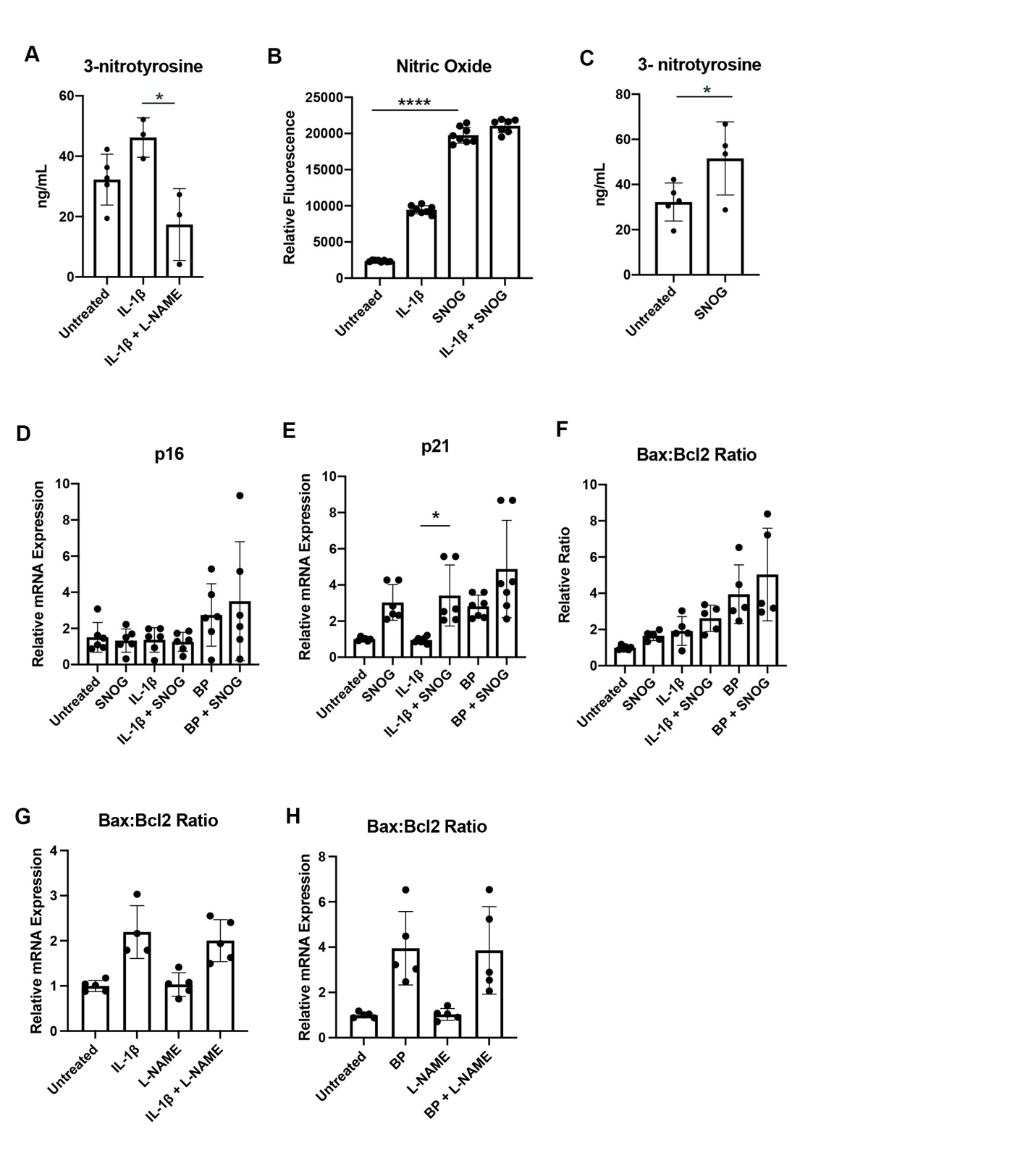
**

**Supplementary Fig S5**. (A) Primary chondrocytes were treated with IL-1β in the presence or absence of L-NAME for 24 hours. 3-Nitrotyrosine levels in the cell were measured by ELISA with n=3-5 replicates (IL-1β vs IL-1β + L-NAME *P=.011). Bars represent mean ± S.D. (B) Chondrocytes were treated with IL-1β in the presence or absence of SNOG (0.6 mM) for 24 hours. Nitric oxide levels were measured by DAF-FM-DA fluorescence (****P<.0001). Bars represent mean ± S.D. for n=8 replicates from one representative experiment. (C) Chondrocytes were treated with SNOG (0.6 mM for 24 hours. 3-Nitrotyrosine levels in the cell were measured by ELISA with n=4-5 replicates (*P=.05). Bars represent mean ± S.D. (D-F) Chondrocytes were treated with IL-1β or BP in the presence or absence of SNOG (0.6 mM) for 24 hours. Gene expression of *Bax*, *Bcl2*, *p16* and *p21* were measured by qPCR. (H: IL-1β vs IL-1β + SNOG *P=.0158). Bars represent mean ± SEM for n=5 independent experiments. (G-H) Chondrocytes were treated with IL-1β or BP in the presence or absence of L-NAME for 24 hours. Gene expression of Bax and Bcl2 was measured and normalized to actin expression. Bax and Bcl2 ratios were then determined, normalizing to untreated cells. Bars are mean ± S.E.M from n=5 independent experiments.
